# Dental aesthetic self-perception, smile satisfaction and associated factors among adults: A cross-sectional study

**DOI:** 10.1371/journal.pone.0357126

**Published:** 2026-09-15

**Authors:** Javaria Baig, Muhammad Osama Afzal, Ramish Mehmood Shaikh, Abdullah Khalid Sagga, Manal Abdulaziz Murad, Fizzah Baig, Layan Khalid Sagga, Mukhtiar Baig

**Affiliations:** 1 Liaquat College of Medicine & Dentistry, Karachi, Pakistan; 2 Tooth Club Dental Practice, Chelmsford, Essex, England; 3 Liaquat College of Medicine & Dentistry, Karachi, Pakistan; 4 Fifth-year student, Faculty of Medicine, Yeditepe University, Istanbul, Turkiye; 5 General Dentist, Jeddah Specialty Dental Center, Ministry of Health, Jeddah, Saudi Arabia; 6 Family and Community Medicine Department, Faculty of Medicine, Rabigh, King Abdulaziz University, Jeddah, Saudi Arabia; 7 Ziauddin Medical College, Ziauddin University, Karachi, Pakistan; 8 Faculty of Medicine, Medical student, King Abdulaziz University, Jeddah, Saudi Arabia; 9 Department of Clinical Biochemistry, Faculty of Medicine, King Abdulaziz University, Jeddah, Saudi Arabia; Universiti Sains Malaysia, MALAYSIA

## Abstract

**Introduction:**

Dental aesthetics self-perception can influence self-confidence and psychosocial well-being, but evidence from Pakistan remains limited. This study examined dental aesthetic self-perception, smile satisfaction, and associated factors among adults in Karachi, Pakistan.

**Methods:**

This cross-sectional study was conducted at Liaquat College of Medicine and Dentistry (LCMD) in Karachi from November 2, 2022, to March 21, 2023. Adults residing in Karachi were recruited through an online convenience sample, with the questionnaire link distributed by email and social media platforms.

**Results:**

Eight hundred fifty-four individuals participated in the study, comprising 276 males (32.3%) and 578 females (67.7%). Overall, 62.8% of participants were satisfied with their smile, whereas 318 (37.2%) expressed dissatisfaction. A total of 595 (69.7%) agreed that a smile can affect mental, emotional, and social well-being, and 665 (77.9%) believed that smiling alleviates stress. Most respondents (542, 63.4%) agreed that tooth color influences the attractiveness of a smile. Additionally, 335 (39.2%) reported always or sometimes avoiding smiling in photographs. Reported reasons included irregular tooth size or shape (25.4%), yellow or stained teeth (22.7%), misaligned teeth (16.1%), gaps between teeth (8.3%), and other reasons (20%). Gender analysis indicated that more females believed a smile influences their cognitive, emotional, and social well-being (p = 0.032) and the influence of tooth color on attractiveness of a smile (p = 0.034). Binary logistic regression found no significant associations between sociodemographic variables and smile satisfaction.

**Conclusion:**

Overall, 62.8% of participants were satisfied with their smiles, and the measured sociodemographic variables were not significantly associated with smile satisfaction. Most participants believed that smiling reduces stress, enhances personality, and reflects confidence. Further studies using probability-based sampling and formally validated instruments are needed to identify clinical, psychological, and contextual factors associated with smile satisfaction.

## Introduction

Dental aesthetic satisfaction (smile satisfaction) is a critical element that can impact a person's self-esteem and overall well-being. In societal relationships, facial features and oral aesthetics are considered extremely important [1]. An individual's subjective perception of their oral aesthetics might impact their self-confidence, contentment, physical well-being, mental state, and interpersonal connections [2–4]. People who invest in dental aesthetics enhance their appearance, psychological health, and sense of happiness.

In the modern era, people generally believe that they should possess attractive facial features to create a pleasing aesthetic impression and be viewed as physically appealing. A study reported that the eyes contribute 25%, mouth 18%, face shape 15%, Skin 12%, and the nose 6% to the overall assessment of facial attractiveness [5]. Several recent studies have shown that self-perceived smile and dental aesthetics correlate with low self-esteem [4,6,7]. Furthermore, the appearance of teeth and smiles can significantly affect individuals’ confidence, social interactions, and quality of life (QoL) [8].

A study found that one of the most common dental concerns affecting satisfaction was inadequate restoration of anterior teeth [9]. Dissatisfaction with one's smile has also been attributed to tooth arrangement and position [2]. Malocclusion, particularly involving the anterior teeth, can negatively affect an individual's personality and self-esteem [10,11]. Perceptions of personal appearance may trigger negative social responses and contribute to a poorer self-image [10, 11]. Untreated dental caries, unaesthetic or stained anterior restorations, and missing anterior teeth may also contribute to dissatisfaction with dental appearance [12,13].

Dental practitioners should understand the public's perception of smiles to better grasp their patients’ needs and wants, which can help them create more effective treatment plans and also help manage patients’ expectations [14]. Although numerous studies have been published on this topic, perceptions of beauty and attraction have rapidly evolved in the social media era. There is a need for a deeper understanding of how individuals perceive their dental aesthetics and how this view relates to their satisfaction with smiling. Self-perceptions of dental aesthetics are highly subjective and vary significantly among individuals. Examining this aspect can uncover how people assess their smiles, which may differ from clinical evaluations to objective measurements. Smile satisfaction is therefore a useful measure of an individual’s overall satisfaction with dental appearance. Understanding these perceptions is important for providing aesthetic treatment within biological constraints [15]. Consequently, this study aimed to examine dental aesthetic self-perception, smile satisfaction, and related sociodemographic factors, including age, gender, marital status, education, and occupation, among a sample of the general adult population in Karachi, Pakistan.

## Methods

### Research design

This cross-sectional study was conducted at Liaquat College of Medicine and Dentistry (LCMD), Karachi, Pakistan, from November 2, 2022, to March 21, 2023. Ethical approval was obtained from the LCMD Institutional Review Board (Reference No. IRB/D-000046/22). Written informed consent was obtained through a clear statement outlining the study's purpose and asking for the participants’ consent before starting the questionnaire. It was clearly specified that their participation would be voluntary, and completing the online questionnaire would be considered their consent to participate in the study and allow for data publication. Furthermore, our data collection procedure closely followed institutional and national ethical standards as well as the Helsinki Declaration. The data were kept completely anonymous to ensure confidentiality.

Participants were recruited from the general population of Karachi through an online convenience sampling strategy. The questionnaire link was distributed by email and social media platforms, including WhatsApp, Facebook, Instagram, and Twitter, and two reminders were also sent at one-week intervals. The researchers also shared the link through classmates, friends, and family contacts. Eligible participants were male and female adults aged 18 years or older who resided in Karachi. Individuals were excluded if they had received orthodontic treatment or had dental or facial injuries, dental or facial conditions, or craniofacial anomalies (such as cleft lip or palate). Sociodemographic characteristics were the independent variables, and smile satisfaction was the dependent variable. The minimum sample size of 386 was calculated before the start of the study using the Raosoft sample size calculator (http://www.raosoft.com/samplesize.html, accessed October 20, 2022) with a 5% margin of error, 95% confidence level, and an estimated Karachi population of 20 million. The achieved sample of 854 exceeded this minimum and improved the precision of the survey's descriptive statistics and generalizability. Generally, estimates and subgroup analysis. However, the larger sample did not eliminate risk of selection bias arising from online convenience sampling.

### Research instrument

The researchers developed an online questionnaire that incorporated selected questions and concepts from two established instruments: the “Aesthetic Component of the Index of Orthodontic Treatment Need (IOTN-AC)” and the “Psychosocial Impact of Dental Aesthetics Questionnaire (PIDAQ)” [16,17]. The complete validated versions of these instruments were not administered.

A pilot survey involving 50 individuals was conducted to assess questionnaire readability and comprehensibility. After the pilot study, the questionnaire was further modified and validated. Two subject-matter experts reviewed the items for content relevance, clarity, and alignment with the study objectives, and the questionnaire was revised based on the pilot feedback and recommendations. The pilot testing and expert review provided evidence of face and content validity; however, reliability coefficients and factor-analytic validation were not conducted for this study. The questionnaire consisted of two sections. The first section comprised questions related to sociodemographic data, whereas the second part focused on dental aesthetics and smiling. The questions had three types of response options: i) “yes” and “no,” ii) “yes,” “no,” and “maybe,” and iii) “yes,” “no,” and “sometimes.” A question about the smile's aesthetic appearance was answered on a five-point Likert scale ranging from extremely attractive to completely unattractive.

### Inclusivity in global research

Additional information regarding the ethical, cultural, and scientific considerations specific to inclusivity in global research is included in the Supporting Information (S3 Checklist in S3 File).

### Statistical analysis

Data were analyzed using IBM Statistical Package for the Social Sciences (SPSS) for Windows, version 26.0 (IBM Corp., Armonk, NY, USA). Categorical data are presented as frequencies and percentages. Chi-square tests were employed to analyze the relationships among different qualitative variables. Binary logistic regression was performed to assess the association between sociodemographic variables and smile satisfaction, which was coded as a dichotomous outcome (satisfied versus not satisfied). Statistical significance was set at a p-value < 0.05.

## Results

Eight hundred fifty-four individuals participated in the study (276 males, 32.3%; 578 females, 67.7%). More than half of the participants (491, 57.5%) were aged 21–23 years. Other participant characteristics are shown in Table 1.

**Table 1:** General characteristics of participants (N = 854).

Variables	Frequencies	Percentages
**Age**		
18-20	152	17.8
21-23	491	57.5
24-26	64	7.5
27-30	67	7.8
30+	80	9.4
**Gender**		
Male	276	32.3
Female	578	67.7
**Marital status**		
Single	688	80.6
Married	156	18.3
Divorced	10	1.1
**Education**		
Bachelors	451	52.8
Intermediate or less	208	24.4
Masters or more	83	9.7
Professional degree	112	13.1
**Occupation**		
Government Job	17	2.0
Healthcare worker	124	14.5
Housewife	35	4.1
Private sector job	138	16.2
Student	493	57.7
Unemployed	47	5.5

Over one-third of the participants (318, 37.2%) were dissatisfied with their smiles, whereas 536 (62.8%) were satisfied. More than two-thirds (595, 69.7%) believed that a smile can affect mental, emotional, and social health, and 665 (77.9%) agreed that it also helps reduce stress. More than half (509, 59.6%) admired other people's smiles, and 251 (29.4%) reported doing so sometimes. A total of 310 (36.3%) had considered changing the way they smile, and 96 (11.2%) reported considering this sometimes. More than two-thirds agreed that people who smile frequently appear more confident (593, 69.4%), and 542 (63.4%) agreed that tooth color influences smile attractiveness. Overall, 335 (39.2%) participants reported always or sometimes avoiding smiling in photographs, whereas 519 (60.8%) did not avoid smiling (Table 2).

**Table 2:** Responses from participants to various questions (N = 854).

Statements	Yes N (%)	No N (%)	Sometimes N (%)
Do you think a smile can affect your mental, emotional & social health?	595(69.7)	107(12.5)	152(17.8)
Do you admire another person's smile?	509(59.6)	94(11)	251(29.4)
Do you ever think to change the way you smile?	310(36.3)	448(52.5)	96(11.2)
Do you avoid smiling when taking pictures?	204(23.9)	519(60.8)	131(15.3)
**Statements**	**Yes** **N (%)**	**No** **N (%)**	**Maybe** **N (%)**
Do you think a good smile can enhance your personality?	745(87.2)	34(4)	75(8.8)
Do you think people who smile regularly are more confident?	593(69.4)	74(8.7)	187(21.9)
Do you think a smile reduce stress?	665(77.9)	77(9)	112(13.1)
Do you think that the color of your teeth decides how attractive your smile is?	542(63.4)	120(14.1)	192(22.5)
**Statement**	**Yes** **N (%)**	**No** **N (%)**	
Are you satisfied with your own smile?	536(62.8)	318(37.2)	

**Fig 1:**
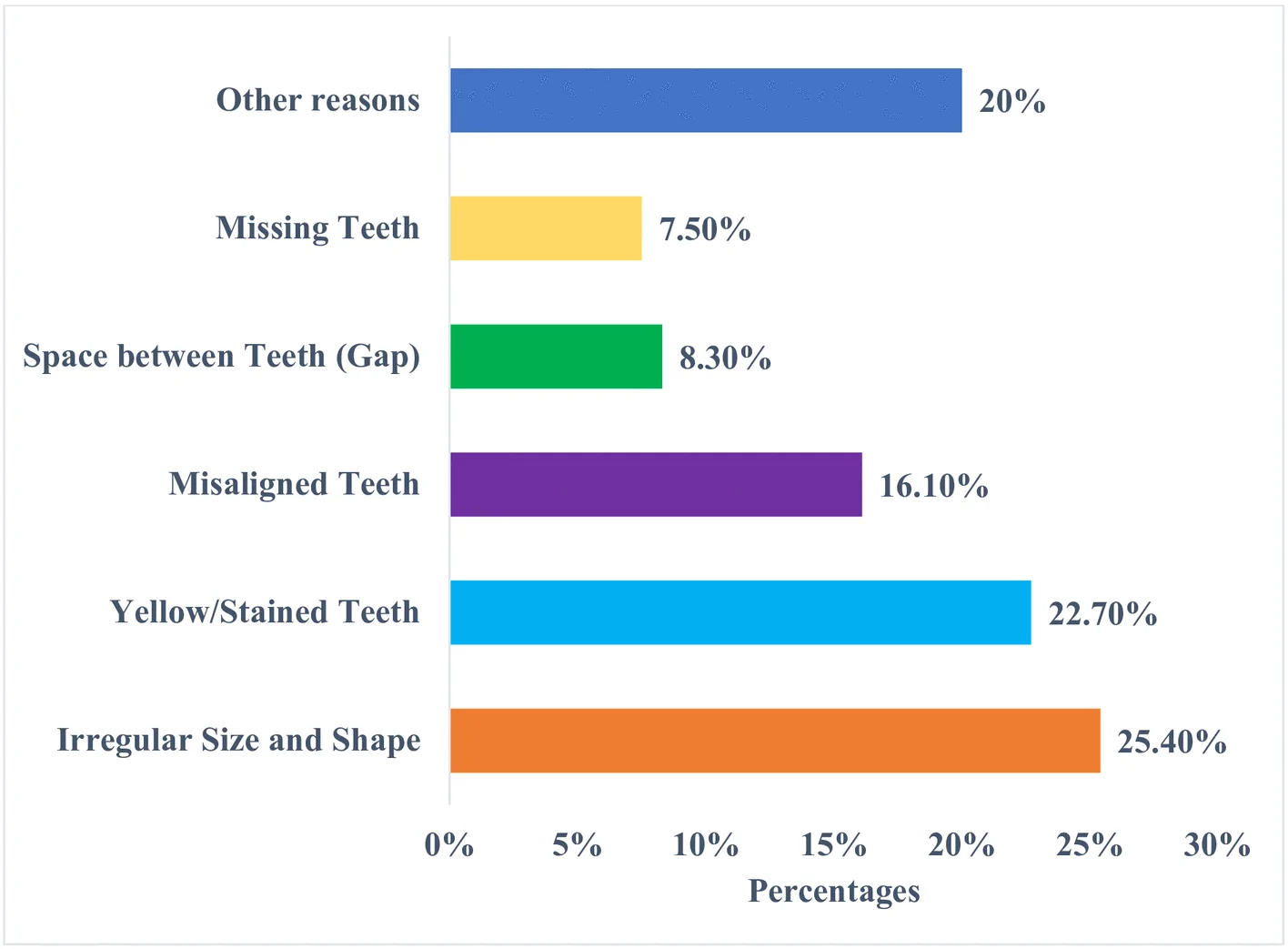
Reasons for avoiding smiling while taking pictures among 335 participants.

Those who avoided smiling while taking pictures had several reasons, as shown in Figure 1.

**Fig 2:**
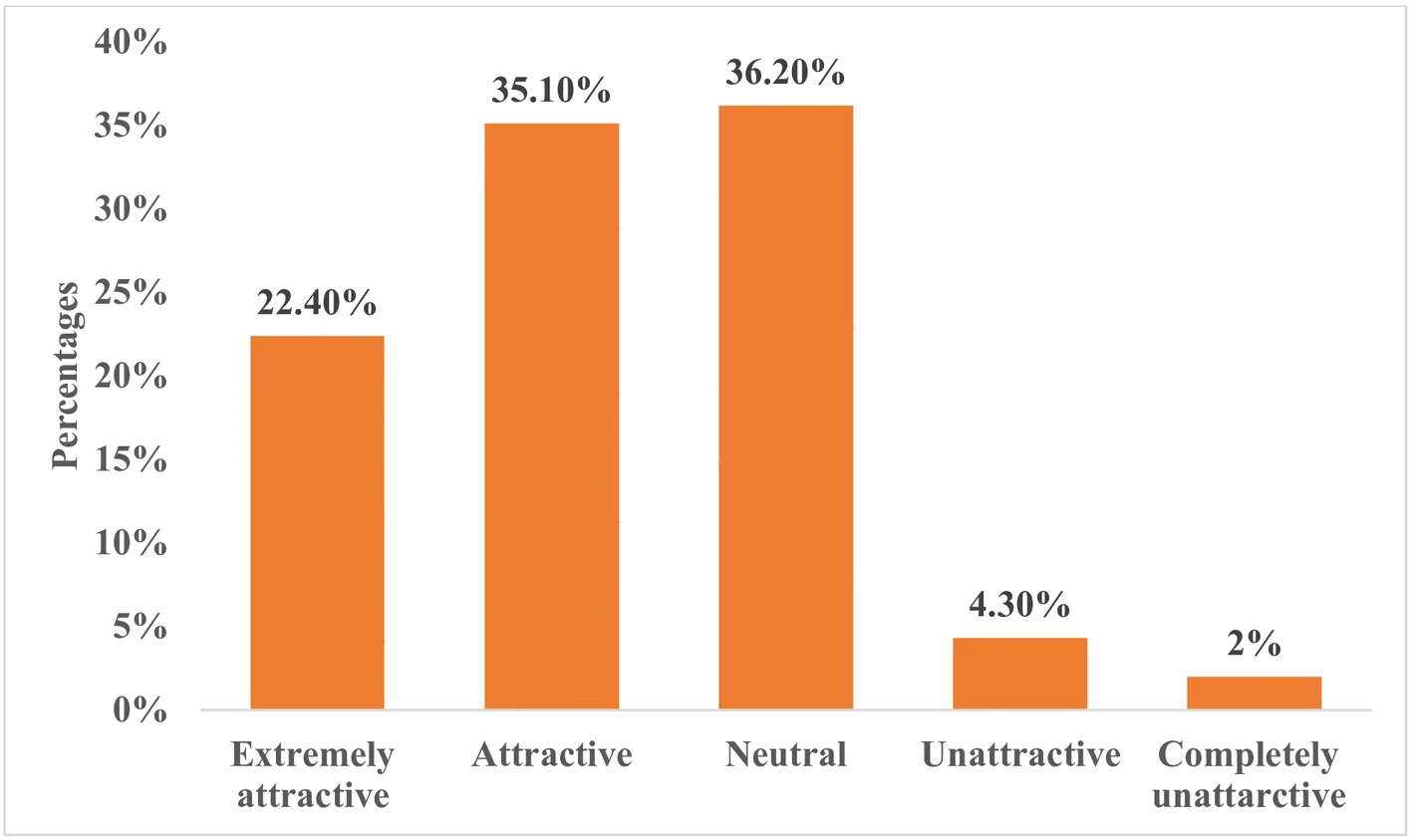
Participants’ responses to the question, “How would you rate the aesthetic appearance of your smile?”.

The participants’ rating of the aesthetic appearance of their smiles is shown in Figure 2.

Gender-stratified response distribution differed significantly for the perceived effect of a smile on mental, emotional, and social well-being (p = 0.032), admiration of another person’s smile (p = 0.033), and the influence of the tooth color on smile attractiveness (p = 0.034). The findings are reported as overall distributional differences because the proportions across the yes, no, and sometimes or maybe categories did not show a consistently higher response in one gender (Table 3).

**Table 3:** Comparison of participant characteristics and questionnaire responses by gender (chi-square test) (N = 854).

Parameters	Responses	Females N(%)	Males N(%)	P value
Age (yrs)	18-20	119(20.6)	33(12)	0.002*
	21-23	335(58)	156(56.5)	
	24-26	41(7.1)	23(8.3)	
	27-30	39(6.7)	28(10.1)	
	30+	44(7.6)	36(13)	
Marital status	Divorced	7(1.2)	3(1.1)	0.796
	Married	109(18.9)	47(17)	
	Single	462(79.9)	226(81.9)	
Education	Bachelors	298(51.6)	153(55.4)	0.001*
	Intermediate	158(27.3)	50(18.1)	
	Masters	43(7.4)	40(14.5)	
	Professional degree	79(13.7)	33(12)	
Occupation:	Government Job	5(0.9)	12(4.3)	< 0.001*
	Healthcare	79(13.7)	45(16.3)	
	Private job	53(9.2)	85(30.8)	
	Students	369(63.8)	124(44.9)	
	Unemployment	37(6.4)	10(3.6)	
Foremost thing you notice when you meet someone for the first time	Smile	281(48.6)	116(42)	0.024*
	Eyes	204(35.3)	127(46)	
	Hair	57(9.9)	20(7.2)	
	Nose	36(3.6)	13(4.7)	
Satisfaction with smile	No	225(38.9)	93(33.7)	0.139
	Yes	353(61.1)	183(66.3)	
Smile affect mental, emotional & social health	No	67(11.6)	40(14.5)	0.032*
	Sometimes	116(20.1)	36(13)	
	Yes	395(68.3)	200(72.5)	
Good smiles enhance the personality	May be	56(9.7)	19(6.9)	0.193
	No	26(4.5)	8(2.9)	
	Yes	496(85.8)	249(90.2)	
Smiling people are confident	Maybe	128(22.1)	59(21.4)	0.417
	No	45(7.8)	29(10.5)	
	Yes	405(70.1)	188(68.1)	
Smile reduces stress	Maybe	80(13.8)	32(11.6)	0.227
	No	46(8)	31(11.2)	
	Yes	452(78.2)	213(77.2)	
Admire another person's smile	No	56(9.7)	38(13.8)	0.033*
	Sometimes	184(31.8)	67(24.3)	
	Yes	338(58.5)	171(62)	
Color of teeth decides attraction of smile	Maybe	142(24.6)	50(18.1)	0.034*
	No	72(12.5)	48(17.4)	
	Yes	364(63)	178(64.5)	
Avoid smiling while taking pictures	No	351(60.7)	168(60.9)	0.998
	Sometimes	89(15.4)	42(15.2)	
	Yes	138(23.9)	66(23.9)	
Thinking to change the smiling way	No	296(51.2)	152(55.1)	0.484
	Sometimes	69(11.9)	27(9.8)	
	Yes	213(36.9)	97(35.1)	
Rating of esthetic appearance of smile	Completely unattractive	11(1.9%)	6(2.2)	0.875
	Unattractive	24(4.2)	13(4.7)	
	Neutral	216(37.4)	93(33.7)	
	Attractive	201(34.8)	99(35.9)	
	Extremely attractive	126(21.8)	65(23.6)	

*P < 0.05 was considered significant.

Binary logistic regression showed no significant association between the measured sociodemographic variables and “smile satisfaction” (Table 4).

**Table 4:** Associations between sociodemographic variables and smile satisfaction among study participants (binary logistic regression).

Parameters vs. smile satisfaction	Responses	Beta Coefficient	Odds Ratio	95% C.I.for Odds Ratio		P-value
Age (yrs)	18-20		Reference	Lower	Higher	
	21-23	−.350	.704	.222	2.234	0.552
	24-26	−.391	.676	.246	1.859	0.448
	27-30	.317	1.373	.411	4.585	0.607
	30+	−.199	.820	.296	2.271	0.702
Gender	Female		Reference			
	Male	−.043	.958	.623	1.473	0.845
Marital status	Divorced		Reference			
	Married	.109	1.115	.190	6.526	0.904
	Single	.104	1.109	.492	2.503	0.803
Education	Graduate		Reference			
	Intermediate	.098	1.103	.606	2.008	0.747
	Masters	.198	1.219	.609	2.440	0.575
	Professional degree	.766	2.152	.827	5.602	0.116
Occupation	Government Job		Reference			
	Healthcare	.516	1.675	.281	10.006	0.571
	Housewives	.167	1.182	.430	3.247	0.746
	Private job	.534	1.707	.431	6.755	0.446
	Students	.454	1.574	.577	4.294	0.376
	Unemployment	.236	1.266	.484	3.315	0.631

* Statistical significance was set at a P < 0.05

*The dependent variable was smile satisfaction (satisfied versus not satisfied).

*Variables entered: gender, age, marital status, education, and occupation.

## Discussion

Smile aesthetics contribute to perceived facial attractiveness, and many individuals seek orthodontic treatment to improve dental appearance. Smiling is an important form of nonverbal communication and has psychological significance [18]. Several features are considered when assessing a smile, including smile pattern, teeth, buccal corridor, midline, gingival display, maxillary incisor exposure, and smile arc [19].

In this study, 37.2% of the participants were dissatisfied with their smiles, whereas 62.8% were satisfied. The satisfaction estimate was similar to findings from Saudi Arabia (66.5%) and Vietnam (67%) [9,20], lower than 80% reported among Saudi adolescents [21], and higher than the 34% reported in Serbia [2]. In contrast, a Malaysian study reported 62.7% dissatisfaction with dental appearance [22]. Changes in smile aesthetics can sometimes lead to reduced self-confidence and self-esteem, making individuals more reserved, withdrawn, and timid [23]. Differences across South Asian, Middle Eastern, Southeast Asian, and European studies may reflect variation in age, cultural context, study population, sampling method, and measurement instrument. The predominance of female and student participants in the present sample may also have influenced the estimates.

The current study revealed no significant differences in dental aesthetic satisfaction between genders. The literature presents conflicting findings on this topic. Some studies indicate that women tend to be less satisfied with their dental aesthetics than men [24,25], while Ghazi et al. (2023) reported higher levels of dental aesthetic satisfaction among females compared to males [4]. In an Indian study, men expressed greater concern about their smiles and reported higher satisfaction with their dental aesthetics than women [26]. It appears that due to changing societal norms and the impact of beauty product companies’ marketing strategies, men are now just as concerned about their physical appearance and aesthetics as women [26]. The differences in our results compared to those of others may stem from varying cultural backgrounds, age disparities, and the use of different questionnaires.

Female respondents most commonly noticed the smile first (48.6%), followed by the eyes (35.3.6%), hair (9.9%), and nose (6.2%) when meeting someone for the first time. In contrast, male respondents most commonly noticed the eyes first (46%), followed by the smile (42%), hair (7.2%), and nose (4.7%). These findings highlight the significance of nonverbal cues during initial social interactions. The observation that smiles and eyes are the two main facial features noticed first underscores the importance of facial expressions in these encounters. It indicates that facial traits associated with positive emotions and friendliness, such as smiling eyes, influence a person's perception in a first meeting. A previous survey reported that teeth were regarded as the most important facial characteristic, while hairlines and ears were viewed as the least significant features [24]. Another study found women scored higher than men on the importance of teeth and hair, but lower on the significance of the head [27]. The observed gender differences in the present study should be interpreted cautiously because of the unequal numbers of female and male participants.

A majority of participants (59.6%) reported admiring another person's smile, supporting the perceived importance of the smile in interpersonal interactions. This finding is consistent with a Saudi study [24]. Overall, 39.2% of participants reported always or sometimes frequently or occasionally avoiding smiling when photographs were taken, with no significant gender difference. Participants provided various reasons for this behavior, including concerns regarding the appearance of their teeth in terms of size, shape, and color, as well as misalignment and gaps between teeth. These findings align with those of previous studies [2,21,28].

Orthodontic or other appropriate dental treatment may address some causes of dissatisfaction with smile. However, the costs of this treatment are high, not only in Karachi but also globally. This poses a significant financial burden for individuals in middle-class and lower-middle-class countries. Consequently, they may go through life feeling unhappy with their smiles. It is crucial to create a plan that ensures access to orthodontic treatment for those with limited financial resources.

Most participants agreed that people who smile frequently appear more confident and that smiling reduces stress. These findings are compelling and highlight the importance of smiling, not only as a sign of happiness but also as a reflection of confidence. Additionally, a smile is associated with positive traits of an individual's personality; similar results have been reported in another study [8].

Approximately two-thirds of participants (63.4%) agreed that tooth color influences smile attractiveness while a further 22.5% responded ‘may be.’ Tooth discoloration can adversely affect a patient's satisfaction and psychological well-being, leading some to choose cosmetic procedures like teeth whitening [29,30]. Overall level of agreement (85.9%) observed in this study regarding the impact of tooth color on smile attractiveness emphasizes the significance of tooth appearance. This suggests that individuals prioritize the beauty and health of their teeth, explaining the high demand for tooth-whitening procedures in modern society.

More than half of the participants (57.5%) rated the aesthetic appearance of their smiles as attractive or extremely attractive, with no significant gender difference. This indicates a generally positive self-rating of smile appearance and is consistent with several previous studies [7,14,25,31]. Facial aesthetics may contribute to perceived well-being, and individuals concerned about their smiles may benefit from professional dental assessment.

This study revealed no associations between age, sex, occupation, education, marital status, or dental aesthetic satisfaction. However, the literature reports conflicting findings [14,27,32,33]. Multiple factors may explain the contrasting results, including a larger proportion of female participants, cultural disparities, a diverse age range in the population sample, and differences in the inclusion and exclusion criteria.

Several confounding factors that we were unable to examine have been emphasized in the literature as having a significant impact on self-perceived dental aesthetics and psychosocial well-being. One study indicated that university affiliation (private vs. public), study discipline, college, and income levels were all significant factors affecting these perceptions [34]. Another study from Saudi Arabia found that “urban residence, tooth color, crowding, unsatisfactory frontal teeth fillings, broken frontal teeth, red inflamed gums, covering teeth while smiling, and the degree of gum display while smiling” were associated with satisfaction regarding dental aesthetics [9]. Furthermore, a study noted that among laypersons, a positive perception of orofacial appearance was related to general health conditions, higher educational levels, and perceptions of oral health conditions [35].

Our research shows that most individuals surveyed agreed on the significant influence a pleasant smile can have on a person's overall demeanor. This further reinforces the idea that an authentic smile expresses happiness and indicates self-assurance. Smiling serves as a powerful symbol of warmth and connection, which is essential to human communication. As a result, dental aesthetics have become increasingly important in modern society, as they can enhance an individual's facial attractiveness [36]. Cosmetic dentistry offers various solutions designed to help individuals achieve their ideal smile, addressing issues such as tooth discoloration, misalignment, and tooth loss [2].

### Implications of the study

The finding that 37.2% of participants were dissatisfied with their smiles supports the value of patient-centered assessment of dental aesthetic concerns. Significant gender differences in several response distributions warrant further investigation, but the unequal gender composition of the sample limits direct interpretation. Longitudinal and mixed-method studies could explore how dental aesthetic concerns relate to psychosocial well-being and why some individuals are dissatisfied with their smiles. Such evidence may inform accessible, clinically appropriate, and patient-centered dental services.

### Limitations

The cross-sectional design precludes causal inference, and all measures were self-reported without comparison with dental records or clinical examinations. The researcher-developed questionnaire incorporated selected content from established instruments and was pilot-tested and reviewed by experts; however, reliability coefficients and factor-analytic validation were not available, which may limit measurement validity and reliability. Online convenience sampling through email, social media, and personal networks may have overrepresented younger, more educated, digitally literate, health-aware, and internet-connected individuals while underrepresenting residents with limited digital access. The sample was also predominantly female (67.7%) and included a large proportion of students, which limits gender comparisons and generalizability. The large achieved sample improves precision but does not remove selection bias. In addition, the study was conducted in one city and did not collect objective clinical data or potentially relevant variables such as tooth shape, tooth color, income, and access to dental care. Recall, social desirability, and other reporting biases are also possible.

## Conclusion

In this sample, the measured sociodemographic variables were not significantly associated with smile satisfaction, and 62.8% of participants reported being satisfied with their smiles. Most participants believed that smiling reduces stress, enhances personality, and conveys confidence. Because important clinical and psychosocial factors were not measured, the absence of sociodemographic associations should not be interpreted as evidence that unmeasured individual experiences are more influential. Further research using representative sampling, validated instruments, and clinical measures is needed to identify factors associated with smile satisfaction and psychosocial well-being.

## Supporting information

S1 File De-identified participant-level dataset underlying the findings reported in this study. (CSV)

S2 File Study questionnaire. (PDF)

S3 File Checklist. (DOCX)
