# Supplementary material for: Dental aesthetic self-perception, smile satisfaction and associated factors among adults: A cross-sectional study
Source: PLoS One. 2026 Sep 15;21(9):e0357126. doi: 10.1371/journal.pone.0357126 (PMC13577522; doi:10.1371/journal.pone.0357126)
Supplement: Supplementary file 1 [file pone.0357126.s002.pdf]

Dental aesthetic self-perception, smile satisfaction and associated factors among adults

1. Gender:

- Female
- Male

2. Age:

- 18-20
- 21-23
- 24-26
- 27-30
- 30+

3. Marital status:

- Single
- Married
- Divorced

4. Education

- Intermediate/ A-levels or less
- Bachelors
- Masters or more
- Professional degree

6. Occupation:

- Student
- Unemployed
- Healthcare worker
- Government Job
- Private Sector Job
- Housewife

7. What is the foremost thing you notice when you meet someone for the first time?

- Eyes

- Nose
- Smile
- Ears

8. Do you think smile can affect your mental, emotional & social health?

- Yes
- No
- Sometimes

9. Do you think a good smile can enhance your personality?

- Yes
- No
- Maybe

10. Do you think people who smile regularly are more confident?

- Yes
- No
- Maybe

11. Do you think smile reduce stress?

- Yes
- No
- Maybe

12. Do you admire another person's smile?

- Yes
- No
- Sometimes

13. Do you think that the color of your teeth decides how attractive your smile is?

- Yes
- No
- Maybe

14. Are you satisfied with your own smile?

- Yes

- No

15. Do you avoid smiling when taking pictures?

- No
- Yes
- Sometimes

16. If yes, then which one of the following is the reason:

- Missing teeth
- Yellow / Stained teeth
- Misaligned teeth
- Irregular size & shape
- Spaces between teeth (Gap)
- Other reason

17. Do you ever think to change the way you smile?

- Yes
- No
- Sometimes

18. If yes? what kind of treatment are you looking for?

- Whitening
- Veneers (artificial covering on teeth)
- Braces
- Replacement
- Denture

19. How would you rate the Esthetic appearance of your smile?

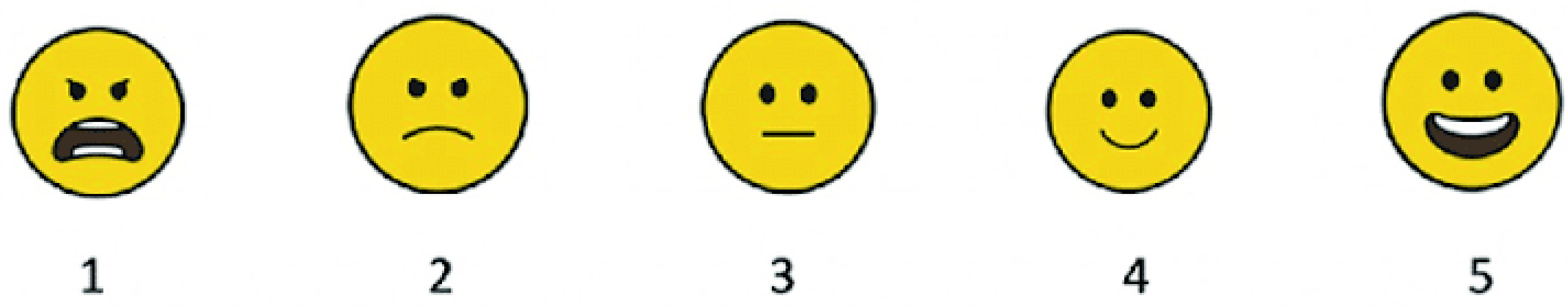

Completely unattractive

1
2
3
4
5

Extremely attractive
