# Supplementary material for: Dental aesthetic self-perception, smile satisfaction and associated factors among adults: A cross-sectional study
Source: PLoS One. 2026 Sep 15;21(9):e0357126. doi: 10.1371/journal.pone.0357126 (PMC13577522; doi:10.1371/journal.pone.0357126)
Supplement: Supplementary file 3 [file pone.0357126.s005.pdf]

Article review Plos one: Dental aesthetic self-perception, smile satisfaction and associated factors among adults: a cross-sectional study

Title and Abstract:

- Title indicated study design
- Gives a brief about what the article is about, significance of the study while the aim is stated clearly.
-

Introduction :

- Line 77 and 78 remove Author initials
- Literature review was not clear on the associated factor impacting the smile perception.
- Rationale clearly stated
- Aim clearly mentioned
- Objectives not stated

Methods:

- Study population not clearly described
- Study setting not explained
- Inclusion and exclusion criteria not clearly stated
- No clear description of how the individual variables were measured, where the study questionnaire was adapted from and was it validated??

Results:

- What was the response rate and explain why
- On the tables, use small letter n, for the study proportions and a small letter p for p value
- General editing required
- No clear mention of the reference group in the regression analysis

Discussion:

- Some statements need references, e.g. line 216 and 218
- There is inconsistency on the mentioned sample size of 386 and the result mention of a total of 864 participants
- Sample size mentioned in the limitations, explain the limitation and mention how to overcome it.
- Statement on line 259, please explain. Did you control for confounding in the regression analysis??

General comments:

- Good study with important findings
- The article needs some editing.
