# Supplementary material for: Dental aesthetic self-perception, smile satisfaction and associated factors among adults: A cross-sectional study
Source: PLoS One. 2026 Sep 15;21(9):e0357126. doi: 10.1371/journal.pone.0357126 (PMC13577522; doi:10.1371/journal.pone.0357126)
Supplement: Supplementary file 4 [file pone.0357126.s009.pdf]

PEER REVIEW REPORT

Manuscript: Dental Aesthetic Self-Perception, Smile Satisfaction , and Associated Factors Among the General Adult Population in Karachi, Pakistan

Study Design: Cross-sectional Survey

Setting: LCMD, Karachi

Period: November 2022-March 2023

Reviewer : Kinder Modisha

1. SUMMARY OF THE MANUSCRIPT

This cross-sectional study investigated dental aesthetic self-perception, smile satisfaction, and associated psychosocial factors in 854 adults recruited from Karachi, Pakistan. Using a self-administered online questionnaire, distributed via email and social media between November 2022 and March 2023. The authors report that 37.2% of participants were dissatisfied with their smiles, that the majority of the participants linked smiling to mental and social well-being, and that there was no significant associations found between sociodemographic variables and smile satisfaction in binary logistic regression.

2. STRENGTHS

- The sample size was adequate (n=854)
- The study addresses clinically and psychologically topics that are relevant, which then will mean that dental care will be patient-centred in their country's context.
- The research question is clearly articulated and the study fills a real gap in the published literature on smile satisfaction in Pakistan population
- Inclusion of gender-disaggregated analysis provides useful insight into different perceptions of smile aesthetics

3. MAJOR CONCERNS

3.1 Sampling Strategy and Selection Bias

With the study relying on convenience sampling via social media and email, it introduces an element of selection bias. Only those with better digital literacy, and stronger health awareness are overrepresented in online surveys. This will be problematic in a city like Karachi, which has a diverse socio-economic landscape, with the lager population group may lack consistent access to internet.

3.2 Absence of a Validated Instrument

The manuscript does not state whether the questionnaire was validated or adapted from an established, validated instrument. Dental aesthetics self-perception research commonly use validated tools such as the Oral Aesthetics Subjective Impact Scale (OASIS)

3.3 Statistical Reporting Errors

Several statistical inconsistencies are present in the abstract and results:

- The p-value for gender analysis of tooth colour is reported as “p<0.034” This is non-standard. If the p value is already below 0.05 then the correct reporting format should be “p=0.034”
- Rounding of percentages is not consistent: the author rounded of 69.67% to 69.7% (to those agreed that smile can impact mental , emotional and social wellbeing. But then rounded off 77,87% to 77,95% which the mumber are not the same

3.4 No mention of Ethical Approval and Informed Consent Documentation

There is no mention of ethical approval from the relevant board or Ethics Committee, nor is there any mention of informed consent procedures for online participation

4. MINOR CONCERNS

4.1 Introduction

The introduction as written in the abstract does not contextualise the study within the existing literature. No reference to any study in relation to smile satisfaction or dental aesthetics

4.2 Methods and Results

- Inclusion and Exclusion criteria are not described
- The gender breakdown is notable way to skew, 32.3% males, 67,7% females

4.3 Discussion And Conclusion

- The claim that more that 50% of subjects expressed contentment with their smiles is inconsistence with the finding that 37.2% were dissatisfied.
- The discussion should compare findings with similar studies from South of Asia, Middle East and other comparable lower-middle income country context

5. REQUIRED ACTIONS AD RECOMMENDATIONS

a) Provide IRB/Ethics Committee approval number and informed consent protocol details
b) Validate or cite the validation status of the Questionnaire used
c) Correct the p-value reporting error (p,0.034 …..p=0.034 and the percentage rounding off)
d) State the inclusion/exclusion criteria clearly in the Methods section
e) Provide a sample size justification or power calculation
f) Discuss the selection criteria bias from internet -based convenience sampling explicitly in the Limitations section
