# Supplementary material for: Dental aesthetic self-perception, smile satisfaction and associated factors among adults: A cross-sectional study
Source: PLoS One. 2026 Sep 15;21(9):e0357126. doi: 10.1371/journal.pone.0357126 (PMC13577522; doi:10.1371/journal.pone.0357126)
Supplement: Supplementary file 6 [file pone.0357126.s006.docx]

**Dear Editor,**

Actually, the present manuscript was rejected by the academic editor without being reviewed. Then we appealed to the journal for resubmission, and the editor allowed us to resubmit. We did not have reviewer comments to reply. However, previous academic editor comments and our response are given below.

**Response to academic editor comments**

**Reason for rejection:** The manuscript addresses a subject already extensively researched and does not add to the current knowledge. Therefore, due to its low priority for publication, my recommendation is that the authors should submit it to a different journal.

**Response:**

We value the editor's comments and appreciate the time taken to assess our work. However, we firmly believe that our manuscript provides valuable insights and warrants a more thorough evaluation by peer reviewers. While it is true that dental aesthetic self-perception has been previously studied, our manuscript contributes to the literature in the following novel ways:

1. **Focus on a diverse and underrepresented population:** Unlike prior studies, our research highlights unique demographic and cultural aspects that influence dental aesthetic self-perception and smile satisfaction. Such findings provide a fresh perspective and have implications for global oral health strategies.
2. **Comprehensive analysis of factors:** We employed a multidisciplinary approach, analyzing not only clinical factors but also psychosocial and behavioral determinants of smile satisfaction. This holistic approach addresses gaps in existing research and paves the way for targeted interventions.
3. **Relevance to the general public and research community:** Dental aesthetics and smile satisfaction are topics of significant interest to both the academic community and the general public. Our study aligns with the journal’s mission to disseminate research that has broad appeal and societal relevance. The findings have the potential to inform public health initiatives and improve patient-centered care in dentistry.

We understand that desk rejections are sometimes necessary to manage editorial priorities. However, we believe that our manuscript addresses important research questions and has the potential to engage your readership. Therefore, we kindly request that the manuscript be reconsidered for review by a new academic editor to ensure an impartial evaluation.

Thank you for considering this request. We are confident that, if given the opportunity, our manuscript will meet the high standards of Plos One. We remain open to revising the manuscript to address any concerns and to ensure it aligns with the journal’s scope and priorities.

**Sincerely,**

**Dr. Javaria Baig**

Liaquat College of Medicine & Dentistry, Karachi, Pakistan

[drjavariabaig@gmail.com](mailto:drjavariabaig@gmail.com), Contact No. 00923312528574
