# Supplementary material for: Dental aesthetic self-perception, smile satisfaction and associated factors among adults: A cross-sectional study
Source: PLoS One. 2026 Sep 15;21(9):e0357126. doi: 10.1371/journal.pone.0357126 (PMC13577522; doi:10.1371/journal.pone.0357126)
Supplement: Supplementary file 7 [file pone.0357126.s007.docx]

Original Article

**Dental aesthetic self-perception, smile satisfaction and associated factors among** **adults: a cross-sectional study**

**Short title**	Dental aesthetic self-perception

**Javaria Baig^1,2*,^ Muhammad Osama Afzal^3^, Ramish Mehmood Shaikh^4^,**

**Abdullah Khalid Sagga^5^, Manal Abdulaziz Murad^6^, Fizzah Baig^7^, Layan Khalid Sagga^8^, Mukhtiar Baig^9^**

^1^ Liaquat College of Medicine & Dentistry, Karachi, Pakistan

**^2^** Tooth Club Dental Practice, Essex, England

^3^ Liaquat College of Medicine & Dentistry, Karachi, Pakistan

^4^ Fifth-year student, Faculty of Medicine, Yeditepe University, Istanbul, Turkiye.

^5^ General Dentist, Jeddah Specialty Dental Center, Ministry of Health, Jeddah, Saudi Arabia

^6^Family and Community Medicine Department, Faculty of Medicine, Rabigh, King Abdulaziz University, Jeddah, Saudi Arabia

**^7^** Ziauddin Medical College, Ziauddin University, Karachi, Pakistan.

**^8^** Medical student, Faculty of Medicine, King Abdulaziz University, Jeddah, Saudi Arabia

^9^ Department of Clinical Biochemistry, Faculty of Medicine, King Abdulaziz University, Jeddah, Saudi Arabia

***Corresponding author**

[drjavariabaig@gmail.com](mailto:drjavariabaig@gmail.com) (JB)

**Abstract**

**Introduction**

The present study examined dental aesthetic self-perception, smile satisfaction, and associated factors among the general adult population in Karachi, Pakistan.

**Methods**

This cross-sectional investigation was conducted at Liaquat College of Medicine and Dentistry (LCMD) in Karachi from November 2, 2022, to March 21, 2023. The online questionnaire link was distributed via email and social media apps.

**Results**

Eight hundred fifty-four individuals participated in the study, comprising 276 males (32.3%) and 578 females (67.7%). Among the participants, 37.2% (318) expressed dissatisfaction with their smiles. When asked whether a smile could impact mental, emotional, and social well-being, 69.7% (595 individuals) agreed, and 77.95% (665 individuals) believed that smiling alleviates stress. Most respondents (63.4%) concurred that the color of a smile affects its attractiveness. Additionally, 39.2% (335) reported avoiding smiling in photographs. Those who refrained from smiling cited various reasons, including irregular tooth size and shape (large/small) (25.4%), yellow/stained teeth (22.7%), misaligned teeth (16.1%), gaps between teeth (8.3%), and others (20%). Gender analysis indicated that more females believed a smile influences their cognitive, emotional, and social well-being (p = 0.032) and that the color of their teeth affects the attractiveness of a smile (p < 0.034). Binary logistic regression analysis found no significant associations between sociodemographic variables and "smile satisfaction."

**Conclusion**

These findings suggest that individual experiences and perceptions are crucial for smile satisfaction, as sociodemographic variables are insignificant factors. Furthermore, more than 50% of the subjects expressed contentment with their smiles. Most participants agreed that a smile reduces stress, enhances personality, and reflects confidence. Further research is necessary to explore the factors associated with dental aesthetic satisfaction and their impact on psychosocial well-being.

**Keywords**

Dental aesthetics; Smile satisfaction; Orthodontics; Psychosocial well-being; Sociodemographic factors

**Introduction**

Dental aesthetic satisfaction (smile satisfaction) is a critical element that can t impact a person's self-esteem and overall well-being. In societal relationships, facial features and oral aesthetics are considered extremely important [1]. An individual's subjective perception of their oral aesthetics might impact their self-confidence, contentment, physicalwell-being, mental state, and interpersonal connections [2-4]. People who invest in dental aesthetics enhance their appearance, psychological health, and sense of happiness.

In the modern era, people generally believe that they should possess attractive facial features to create a pleasing aesthetic impression and be viewed as physically appealing. The eyes, smile, and nose are the most prominent facial features of an individual. Several recent studies have shown that self-perceived smiles and dental aesthetics correlate with adolescent self-esteem [4-6]. Furthermore, the appearance of adults' teeth and smiles can significantly affect their confidence, social interactions, and quality of life (QoL) [7]. Various elements of a smile, such as smile patterns, teeth, buccal strip, midline, gingival display, maxillary incisor exposure, smile arc, and others, are taken into account when assessing a smile [8].

A study that the most common dental concerns affecting people's satisfaction was inadequate filling of front teeth [9]. Dissatisfaction with one's smile was primarily attributed to the arrangement and positioning of teeth [2]. Malocclusion particularly in the front teeth, negatively impacts an individual's personality and self-esteem [10,11], The way individuals perceive their appearance significantly influences their personality. and can trigger negative social responses that mayultimately lead to a reduced self-image [10,11]. Teeth may be undermined the the presence of: untreated dental caries, unsightly or stained restorations on the front teeth, and missing front teeth often contribute to dissatisfaction with dental appearance [12,13].

Dental practitioners should understand the public's perception of smiles to better grasp their patients' needs and wants, which can help them create more effective treatment plans [14]. Although numerous studies have been published on this topic, perceptions of beauty and attraction have rapidly evolved in the social media era. There is a need for a deeper understanding of how individuals perceive their dental aesthetics and how this view relates to their satisfaction with smiling. Self-perceptions of dental aesthetics are highly subjective and vary significantly among individuals. Examining this aspect can uncover how people assess their smiles, which may differ from clinical evaluations to objective measurements. Smile satisfaction is a key metric that gauges a person's overall happiness with their dental appearance. The literature emphasizes that understanding individuals' perceptions of dental aesthetics is essential if their needs and effectively addressing their expectations for aesthetic therapy [15]. Consequently, this study aimed to explore dental aesthetic self-perception, smile satisfaction, and related factors among a sample of the adult general population in Karachi, Pakistan.

**Methods**

Research design

The present cross-sectional investigation was conducted at Liaquat College of Medicine and Dentistry (LCMD) in Karachi, Pakistan, from November 2, 2022, to March 21, 2023. Ethical approval was obtained from the LCMD institutional review board (Reference No. IRB/D-000046/22). Written informed consent was obtained through a clear statement outlining the study's purpose and asking for the participants' consent before starting the questionnaire. It was clearly specified that their participation would be voluntary, and completing the online questionnaire would be considered their consent to participate in the study and allow for data publication. Furthermore, our data collection procedure closely followed institutional and national ethical standards as well as the Helsinki Declaration. The data were kept completely anonymous to ensure the preservation of confidentiality.

The online questionnaire link was distributed via email and social media platforms, including WhatsApp, Facebook, Instagram, and Twitter. Additionally, the researchers used the participants' friends and family contacts to reach out to them. The independent variable in this research was sociodemographic characteristics, while smile satisfaction served as the dependent variable. The study included male and female volunteers who were residents of Karachi. The exclusion criteria included individuals who had received orthodontic treatment or had dental or facial issues, injuries, or craniofacial anomalies (such as cleft lip or palate). The sample size, 386, was determined before the start of the study using the Raosoft sample size calculator. The data were collected using a convenience sampling technique.

**Research instrument**

The researchers designed an online questionnaire and included a few questions from already published well-known questionnaires, namely, the "Aesthetic Component (AC) of the Index of Orthodontic Treatment Need (IOTN)" and the "Psychosocial Impact of Dental Aesthetics Questionnaire (PIDAQ)" [16,17]. Two subject experts checked the content and construct validity of the questionnaire, and modifications were made according to their suggestions.

A pilot survey was conducted with 50 individuals to assess questionnaire readability and understandability. After the pilot study, the questionnaire was further modified . The questionnaire consisted of two sections. The first section comprised questions related to sociodemographic data, whereas the second part focused on dental aesthetics and smiling. The questions had three types of response options: i) "yes" and "no," ii) "yes," "no," and "maybe," and iii) "yes," "no," and "sometimes." A question about the smile's aesthetic appearance was answered on a five-point Likert scale ranging from extremely attractive to completely unattractive.

**Statistical analysis**

The data were analyzed using SPSS-26. The qualitative data are presented as frequencies Percentages were calculated, and a chi-square test was employed to analyze the relationships among different. qualitative variables. Binary logistic regression analysis was performed to identify predictors of smile satisfaction, considering it a dichotomous variable (either satisfied or not satisfied). The significance level was set at a p-value < 0.05.

**Results**

Eight hundred fifty-four individuals participated in the study (276 males, 32.3%; 578 females, 67.7%). More than half of the participants, 491 (57.5%), were in the 21-23. Other characteristics are shown in Table 1.

**Table 1.** General characteristics of participants (N=854).

| **Variables** | **Frequencies** | **Percentages** |
| --- | --- | --- |
| **Age** | | |
| 18-20 | 152 | 17.8 |
| 21-23 | 491 | 57.5 |
| 24-26 | 64 | 7.5 |
| 27-30 | 67 | 7.8 |
| 30+ | 80 | 9.4 |
| **Gender** | | |
| Male | 276 | 32.3 |
| Female | 578 | 67.7 |
| **Marital status** | | |
| Single | 688 | 80.6 |
| Married | 156 | 18.3 |
| Divorced | 10 | 1.1 |
| **Education** | | |
| Bachelors | 451 | 52.8 |
| Intermediate or less | 208 | 24.4 |
| Masters or more | 83 | 9.7 |
| Professional degree | 112 | 13.1 |
| **Occupation** | | |
| Government Job | 17 | 2.0 |
| Healthcare worker | 124 | 14.5 |
| Housewife | 35 | 4.1 |
| Private sector job | 138 | 16.2 |
| Student | 493 | 57.7 |
| Unemployed | 47 | 5.5 |

Over one-third of the participants, 318 (37.2%), were dissatisfied with their smiles, while 536 (62.8%) were satisfied. In response to one question, more than two-thirds, 595 (69.7%), believed that a smile can impact one's mental, emotional, and social health, and 665 (77.95%) agreed that it also helps reduce stress. More than half of the participants, 509 (59.6%), admired other people's smiles, and approximately one-third, 251 (29.4%), indicated this occasionally. More than one-third of the participants, 310 (36.3%), felt that they might alter the way they smile, and 96 (11.2%) believed that they would sometimes change their smile. Over two-thirds of the participants agreed that people who smile frequently appear more confident (593, 69.4%), and 542 (63.4%) agreed that the color of teeth influences the attractiveness of a smile. Regarding the tendency to avoid smiling in photographs, approximately 335 (39.2%) participants responded positively or sometimes, while 519 (60.8%) said they do not avoid smiling (Table 2).

**Table 2.** Responses from participants to various questions (N=854).

| **Statements** | **Yes**  **N(%)** | **No**  **N(%)** | **Sometimes**  **N(%)** |
| --- | --- | --- | --- |
| Do you think a smile can affect your mental, emotional & social health? | 595(69.7) | 107(12.5) | 152(17.8) |
| Do you admire another person's smile? | 509(59.6) | 94(11) | 251(29.4) |
| Do you ever think to change the way you smile? | 310(36.3) | 448(52.5) | 96(11.2) |
| Do you avoid smiling when taking pictures? | 204(23.9) | 519(60.8) | 131(15.3) |
| **Statements** | **Yes**  **N(%)** | **No**  **N(%)** | **Maybe**  **N(%)** |
| Do you think a good smile can enhance your personality? | 745(87.2) | 34(4) | 75(8.8) |
| Do you think people who smile regularly are more confident? | 593(69.4) | 74(8.7) | 187(21.9) |
| Do you think a smile reduce stress? | 665(77.9) | 77(9) | 112(13.1) |
| Do you think that the color of your teeth decides how attractive your smile is? | 542(63.4) | 120(14.1) | 192(22.5) |
| **Statement** | **Yes**  **N(%)** | **No**  **N(%)** |  |
| Are you satisfied with your own smile? | 536(62.8) | 318(37.2) |  |

Those who avoided smiling while taking pictures had several reasons shown in Figure 1.

**Fig 1. Reasons for avoiding smiling while taking pictures among 335 participants.**

The participants’ rating of the aesthetic appearance of their smiles is shown in Figure 2.

**Fig 2. Participants' responses to the question, “How would you rate the aesthetic appearance of your smile?”**

Gender-wise analysis revealed that more females thought that dental aesthetics affect their cognitive, emotional, and social well-being (p=.032) and that the color of their teeth might determine the attraction of a smile (p< 0.034). More females than males admire other people's smiles (p< 0.033) (Table 3).

**Table 3.** Comparison of participants' general characteristics by gender (chi-square test) (N = 854).

| Parameters | Responses | Females  N(%) | Males  N(%) | P value |
| --- | --- | --- | --- | --- |
| Age (yrs) | 18-20 | 119(20.6) | 33(12) | 0.002* |
|  | 21-23 | 335(58) | 156(56.5) |  |
|  | 24-26 | 41(7.1) | 23(8.3) |  |
|  | 27-30 | 39(6.7) | 28(10.1) |  |
|  | 30+ | 44(7.6) | 36(13) |  |
| Marital status | Divorced | 7(1.2) | 3(1.1) | 0.796 |
|  | Married | 109(18.9) | 47(17) |  |
|  | Single | 462(79.9) | 226(81.9) |  |
| Education | Bachelors | 298(51.6) | 153(55.4) | 0.001* |
|  | Intermediate | 158(27.3) | 50(18.1) |  |
|  | Masters | 43(7.4) | 40(14.5) |  |
|  | Professional degree | 79(13.7) | 33(12) |  |
| Occupation: | Government Job | 5(0.9) | 12(4.3) | < 0.001* |
|  | Healthcare | 79(13.7) | 45(16.3) |  |
|  | Private job | 53(9.2) | 85(30.8) |  |
|  | Students | 369(63.8) | 124(44.9) |  |
|  | Unemployment | 37(6.4) | 10(3.6) |  |
| Foremost thing you notice when you meet someone for the first time | Smile | 281(48.6) | 116(42) | 0.024* |
|  | Eyes | 204(35.3) | 127(46) |  |
|  | Hair | 57(9.9) | 20(7.2) |  |
|  | Nose | 36(3.6) | 13(4.7) |  |
| Satisfaction with smile | No | 225(38.9) | 93(33.7) | 0.139 |
|  | Yes | 353(61.1) | 183(66.3) |  |
| Smile affect mental, emotional & social health | No | 67(11.6) | 40(14.5) | 0.032* |
|  | Sometimes | 116(20.1) | 36(13) |  |
|  | Yes | 395(68.3) | 200(72.5) |  |
| Good smiles enhance the personality | May be | 56(9.7) | 19(6.9) | 0.193 |
|  | No | 26(4.5) | 8(2.9) |  |
|  | Yes | 496(85.8) | 249(90.2) |  |
| Smiling people are confident | Maybe | 128(22.1) | 59(21.4) | 0.417 |
|  | No | 45(7.8) | 29(10.5) |  |
|  | Yes | 405(70.1) | 188(68.1) |  |
| Smile reduces stress | Maybe | 80(13.8) | 32(11.6) | 0.227 |
|  | No | 46(8) | 31(11.2) |  |
|  | Yes | 452(78.2) | 213(77.2) |  |
| Admire another person's smile | No | 56(9.7) | 38(13.8) | 0.033* |
|  | Sometimes | 184(31.8) | 67(24.3) |  |
|  | Yes | 338(58.5) | 171(62) |  |
| Color of teeth decides attraction of smile | Maybe | 142(24.6) | 50(18.1) | 0.034* |
|  | No | 72(12.5) | 48(17.4) |  |
|  | Yes | 364(63) | 178(64.5) |  |
| Avoid smiling while taking pictures | No | 351(60.7) | 168(60.9) | 0.998 |
|  | Sometimes | 89(15.4) | 42(15.2) |  |
|  | Yes | 138(23.9) | 66(23.9) |  |
| Thinking to change the smiling way | No | 296(51.2) | 152(55.1) | 0.484 |
|  | Sometimes | 69(11.9) | 27(9.8) |  |
|  | Yes | 213(36.9) | 97(35.1) |  |
| Rating of esthetic appearance of smile | Completely  unattractive | 11(1.9%) | 6(2.2) | 0.875 |
|  | Unattractive | 24(4.2) | 13(4.7) |  |
|  | Neutral | 216(37.4) | 93(33.7) |  |
|  | Attractive | 201(34.8) | 99(35.9) |  |
|  | Extremely attractive | 126(21.8) | 65(23.6) |  |

*P < 0.05 was considered significant.

The binary logistic regression analysis revealed that no sociodemographic variables were associated with “smile satisfaction" (Table 4).

**Table 4.** Association of variables with satisfaction related to smiles among study participants (binary logistic regression analysis).

| Parameters vs. smile satisfaction | Responses | Beta Coefficient | Odds Ratio | 95% C.I.for Odds Ratio | | P-value |
| --- | --- | --- | --- | --- | --- | --- |
| Age (yrs) | 18-20 |  | Reference | Lower | Higher |  |
|  | 21-23 | -.350 | .704 | .222 | 2.234 | 0.552 |
|  | 24-26 | -.391 | .676 | .246 | 1.859 | 0.448 |
|  | 27-30 | .317 | 1.373 | .411 | 4.585 | 0.607 |
|  | 30+ | -.199 | .820 | .296 | 2.271 | 0.702 |
| Gender | Female |  | Reference |  |  |  |
|  | Male | -.043 | .958 | .623 | 1.473 | 0.845 |
| Marital status | Divorced |  | Reference |  |  |  |
|  | Married | .109 | 1.115 | .190 | 6.526 | 0.904 |
|  | Single | .104 | 1.109 | .492 | 2.503 | 0.803 |
| Education | Graduate |  | Reference |  |  |  |
|  | Intermediate | .098 | 1.103 | .606 | 2.008 | 0.747 |
|  | Masters | .198 | 1.219 | .609 | 2.440 | 0.575 |
|  | Professional degree | .766 | 2.152 | .827 | 5.602 | 0.116 |
| Occupation: | Government Job |  | Reference |  |  |  |
|  | Healthcare | .516 | 1.675 | .281 | 10.006 | 0.571 |
|  | Housewives | .167 | 1.182 | .430 | 3.247 | 0.746 |
|  | Private job | .534 | 1.707 | .431 | 6.755 | 0.446 |
|  | Students | .454 | 1.574 | .577 | 4.294 | 0.376 |
|  | Unemployment | .236 | 1.266 | .484 | 3.315 | 0.631 |

*Binary logistic regression analysis was applied at a P value < 0.05

*The dependent variable is smile satisfaction, i.e., satisfied or not satisfied

*Variable(s): Gender, Age, Marital status, Education, Occupation.

**Discussion**

The aesthetics of a smile play a crucial role in enhancing facial beauty, and many individuals seek orthodontic treatment to improve the appearance of their teeth. Smiling is a powerful way to connect with others, even without words, and it holds significant psychological importance.

In this study, over one-third of the participants expressed dissatisfaction with their smiles. This group likely has specific concerns or insecurities regarding the alignment, color, or overall appearance of their teeth, as well as other dental and facial features. Approximately two-thirds of the participants reported being content with their smiles, perceiving them as authentic and effective for social interactions and self-presentation. These findings are consistent with studies conducted in Saudi Arabia and Vietnam, which found that most participants were satisfied with their teeth and smiles [9,19].

Conversely, a recent study in Serbia reported a significantly lower satisfaction rate among participants (34%) [2], while a study in Saudi Arabia reported a much higher satisfaction rate (80%) [20]. Changes in smile aesthetics can sometimes lead to reduced self-confidence and self-esteem, making individuals more reserved, withdrawn, and timid [21]. Various factors may contribute to inconsistent outcomes. Some studies only involved dental students, whereas our study included a diverse population from different age groups. Moreover, the present study had a larger proportion of female participants, which could be a significant factor influencing the variability in our findings.

The current study revealed no significant differences in dental aesthetic satisfaction between genders. The literature presents conflicting findings on this topic. Some studies indicate that women tend to be less satisfied with their dental aesthetics than men [22,23], while Ghazi et al. (2023) reported higher levels of dental aesthetic satisfaction among females compared to males. In an Indian study, men expressed greater concern about their smiles and reported higher satisfaction with their dental aesthetics than women [24]. It appears that due to changing societal norms and the impact of beauty product companies' marketing strategies, men are now just as concerned about their physical appearance and aesthetics as women [24]. The differences in our results compared to those of others may stem from varying cultural backgrounds, age disparities, and the use of different questionnaires.

The present study revealed that female respondents tend to notice the smile first, followed by the eyes, hair, and nose when meeting someone for the first time. In contrast, males primarily notice the eyes first, followed by smiles, hair, and noses. This outcome highlights the significance of nonverbal cues during initial social interactions. The observation that smiles and eyes are the two main facial features noticed first underscores the importance of facial expressions in these encounters. It indicates that facial traits associated with positive emotions and friendliness, such as smiling eyes, influence a person's perception in a first meeting. A survey showed that teeth were regarded as the most important facial characteristic, while hairlines and ears were viewed as the least significant features. In another study, women scored higher than men on the importance of teeth and hair, but lower on the significance of the head. The female participants' preference for smiles suggests that women are more attuned to the aesthetic sense of beauty and place greater emphasis on smiles compared to men.

A significant majority of the participants expressed admiration for another person's smile. This finding highlights recognition and of the positive effect of a smile on interpersonal interactions. The results parallel those of a Saudi study [22]. Thirty-nine percent of participants reported frequently or occasionally avoiding smiling when their photographs were taken; therewere no gender disparities . Participants provided various reasons for this behavior, including concerns regarding the appearance of their teeth in terms of size, shape, and color, as well as misalignments and gaps. These findings align with those of previous studies [2,20,26].

One possible solution to address dissatisfaction with smiles is orthodontic therapy. However, the costs of this treatment are high, not only in Karachi but also globally. This poses a significant financial burden for individuals in middle-class and lower-middle-class countries, . Consequently, they may go through life feeling unhappy with their smiles. It is crucial to create a plan that ensures access to orthodontic treatment for those with limited financial resources.

The data also revealed that most participants agreed that people who smile frequently are seen as more confident and experience less stress. These findings are compelling and highlight the importance of a genuine smile, not only as a sign of happiness but also as a reflection of confidence. Additionally, a smile is associated with positive traits of an individual's personality; similar results have been reported in another study [7].

The current data indicate that roughly two-thirds of the participants agreed that the color of their teeth significantly influences smile attractiveness. Tooth discoloration can adversely affect a patient's satisfaction and psychological well-being, leading some to choose cosmetic procedures like teeth whitening [27,28]. The level of agreement observed in this study regarding the impact of tooth color on smile attractiveness emphasizes the significance of tooth appearance. This suggests that individuals prioritize the beauty and health of their teeth, explaining the high demand for tooth-whitening procedures in modern society.

More than half of the participants in this study rated the aesthetic appearance of their smiles as either extremely attractive or attractive, with no significant gender differences. This indicates a high level of self-confidence and a positive self-image regarding their smiles. These results are consistent with those of several other studies [6,14,23,30]. Facial aesthetics play an important role in individuals' perception of their well-being. If any issues arise related to one's smile, it is wise to consult a dentist for professional advice.

This study revealed no associations between age, sex, occupation, education, marital status, or dental aesthetic satisfaction. However, the literature reports conflicting findings [14,25,31,32]. Multiple factors may explain the contrasting results, including a larger proportion of female participants, cultural disparities, a diverse age range in the population sample, and differences in the inclusion and exclusion criteria.

Several confounding factors that we were unable to examine have been emphasized in the literature as having a significant impact on self-perceived dental aesthetics and psychosocial well-being. One study indicated that university affiliation (private vs. public), study discipline, college, and income levels were all significant factors affecting these perceptions [33]. Another study from Saudi Arabia found that “urban residence, tooth color, crowding, unsatisfactory frontal teeth fillings, broken frontal teeth, red inflamed gums, covering teeth while smiling, and the degree of gum display while smiling” were associated with satisfaction regarding dental aesthetics [9]. Furthermore, a study noted that among laypersons, a positive perception of orofacial appearance was related to general health conditions, higher educational levels, and perceptions of oral health conditions [34].

Our research shows that most individuals surveyed agreed on the significant influence a pleasant smile can have on a person's overall demeanor. This further reinforces the idea that an authentic smile expresses happiness and indicates self-assurance. Smiling serves as a powerful symbol of warmth and connection, which is essential to human communication. As a result, dental aesthetics have become increasingly important in modern society, as they can enhance an individual's facial attractiveness. Cosmetic dentistry offers various solutions designed to help individuals achieve their ideal smile, addressing issues such as tooth discoloration, misalignment, and tooth loss.

**Implications of the study**

Our results indicate several exciting findings. Many study participants expressed dissatisfaction with their smiles and desired to improve them. Considering their social and emotional needs, it is essential to promote positive attitudes toward smiles among these individuals. The current study also revealed gender differences in the perception of smiles, highlighting the need for gender-specific healthcare and marketing strategies. Additionally, the influence of smiles on mental and emotional well-being underscores the necessity of integrating smile-focused treatments into comprehensive wellness programs. The findings of this study suggest a need for longitudinal mixed-method studies that involve people in in-depth conversations to understand their perceptions and the reasons behind their dissatisfaction with their smiles. Our findings have significant implications for all stakeholders.

**Limitations**

As a cross-sectional study, it is not possible to establish a causal relationship. The findings of this study may be limited by their reliance on self-reported information from respondents. The researchers did not compare individuals' self-perceived dental issues to their dental records or conduct expert examinations. Additionally, the sample size and demographic composition, particularly the higher proportion of females, may impact the generalizability of the results to larger populations. Despite their significant influence on self-perceived satisfaction, data on tooth shape and color were not collected. Furthermore, convenience sampling from a single city may introduce sampling bias, and participant reporting biases must be acknowledged in this study.

**Conclusion**

According to the results of the regression analysis, our findings suggest that sociodemographic variables are not significantly associated with dental aesthetic satisfaction. Instead, they indicate that individual experiences and perceptions play a more influential role in determining dental aesthetic satisfaction. Most subjects expressed satisfaction with their smiles. During their initial encounters, the participants noted two key facial features: smiles and eyes. Most agreed that a smile can reduce stress, enhance personality, and convey confidence. These findings highlight the need to address concerns related to smiles and promote a positive attitude toward them for overall well-being and self-confidence. Further research is needed to identify the factors associated with satisfaction regarding smiles and their impact on psychosocial wellness.

**Statements and declarations**

**Competing interests**

No competing interests

**Funding**

 This study was not funded by any institute/agency/pharmaceutical company.

**Supporting information**

S1 File. Study dataset.

**Acknowledgments**

The authors thank all the study participants for their cooperation.

**References**

1. Subramani P, Neelakantan S, Johnson SP, Srinivasan S. Association between Confidence in Smiling and Esthetic Characteristics: A Cross-sectional Study in Chengalpattu. J Sci Dent. 2021;11:16-8.
2. Stojilković M, Gušić I, Berić J, Prodanović D Pecikozić N, Veljović T, et al. Evaluating the influence of dental aesthetics on psychosocial well-being and self-esteem among students of the University of Novi Sad, Serbia: a cross-sectional study. BMC Oral Health. 2024;24:277.
3. Tu MH, Gilbert EK, Bono JE. Is beauty more than skin deep? Attractiveness, power, and nonverbal presence in evaluations of hirability. Pers Psychol. 2022;75:119-46.
4. Ghazi OM, Alhashimi AH, Jasim GM. Assessment of the psychological impact of dental aesthetics among undergraduate university students in Iraq. J Orthodont Sci. 2023;12:71-7.
5. Akpasa IO, Yemitan TA, Ogunbanjo BO, Oyapero A. Impact of severity of malocclusion and self-perceived smile and dental aesthetics on self-esteem among adolescents. J World Fed Orthod. 2022;11:120-4.
6. Gavic L, Budimir M, Tadin A. The association between self-esteem and aesthetic component of smile among adolescents. Prog Orthod. 2024;25:9.
7. Isiekwe GI, Sofola OO, Onigbogi OO, Utomi IL, Sanu OO. Dental esthetics and oral health-related quality of life in young adults. Am J Orthod Dentofacil Orthop. 2016;150:627-36.
8. Xiao Z, Chen G, Zhao Y, Wang Y, Gu Y. Perceptual difference of smile aesthetics between 2-dimensional photographs and 3-dimensional dentofacial images: a cross-sectional study. BMC Oral Health. 2023;23:104.
9. Abouzeid HL, Mustafa MM, Alqahtani SM, Tasleem R, Gadah TS, Elmahdy AE. Saudi Self-Concept, Attitude and Factors Influencing Their Satisfaction toward Dental Appearance- an Online Questionnaire Survey. Ann Med Health Sci Res. 2022;13: 413-9
10. Nazir R, Mahmood A, Ayesha A. Assessment of psychosocial impact of dental aesthetics and self perceived orthodontic treatment need in young adults. Pak Oral Dent J. 2014;34:312-6.
11. Gassem AA, Aldweesh AH, Alsagob EI, Alanzi AM, Hafiz MA, Aljohani SR et al. Psychosocial impact of malocclusion and self-perceived orthodontic treatment need among young adult dental patients. Eur J Dent. 2022;17:713-9.
12. Shah RJ, Malek FG, Agarwal P. A study of patient satisfaction with maxillary anterior teeth restorations and desirable esthetic treatment options. J Dent Med Sci. 2014;13:79-86.
13. Al-Omiri M, Karasneh J, Lynch E, Lamey P, Clifford T. Impacts of missing upper anterior teeth on daily living. Int Dent J. 2009;59:127-32.
14. Mannaa AI. Knowledge and Attitude Toward Esthetic Dentistry and Smile Perception. Cureus. 2023;15: e46043.
15. Armalaite J, Jarutiene M, Vasiliauskas A, Sidlauskas A, Svalkauskiene V, Sidlauskas M, et al. Smile aesthetics as perceived by dental students: A cross‑sectional study. BMC Oral Health. 2018;18:225.
16. Brook PH, Shaw WC. 1989. The development of an index of orthodontic treatment priority. Eur J Orthod. 11: 309–320.
17. Klages U, Claus N, Wehrbein H, Zentner A. Development of a questionnaire for assessment of the psychosocial impact of dental aesthetics in young adults. Eur J Orthod 2006;28:103–11.
18. Badran SA. The effect of malocclusion and self-perceived aesthetics on the self-esteem of a sample of Jordanian adolescents. Eur J Orthod. 2010;32:638-44.
19. Nguyen VT, Tran NN, Nguyen NH, Nguyen TD. Evaluating smile aesthetic satisfaction and related smile characteristics in dental students. J Oral Biol Craniofac Res. 2024;14:92-7.
20. Ellakany P, Fouda SM, Alghamdi M, Bakhurji E. Factors affecting dental self-confidence and satisfaction with dental appearance among adolescents in Saudi Arabia: a cross-sectional study. BMC Oral Health. 2021; 21: 149.
21. Silva IF, Paschoal MA, Nascimento AD, Lago AD. Perception of social and emotional characteristics through the smile. Res Soc Dev. 2022;11:e21111326326.
22. Al-Saleh S, Abu-Raisi S, Almajed N, Bukhary F. Esthetic self-perception of smiles among a group of dental students. Int J Esthet Dent. 2018;13:220-30.
23. da Silva GD, de Castilhos ED, Masotti AS, Rodrigues-Junior SA. Dental esthetic self-perception of Brazilian dental students. Rev Sul-Bras Odontol. 2012;9(4):375-81.
24. Afroz S, Rathi S, Rajput G, Rahman SA. Dental esthetics and its impact on psychosocial well-being and dental self-confidence: a campus-based survey of north Indian university students. J Indian Prosthodont Soc. 2013;13:455-60.
25. Jørnung J, Fardal Ø. Perceptions of patients' smiles: a comparison of patients' and dentists' opinions. J Am Dent Assoc. 2007;138:1544-53.
26. Isiekwe GI, Aikins EA. Self-perception of dental appearance and aesthetics in a student population. Int Orthod. 2019;17:506-12.
27. Bersezio C, Martín J, Mayer C, et al. Quality of life and tooth color stability change three months after dental bleaching. Qual Life Res. 2018;27:3199–207.
28. Samorodnitzky-Naveh GR, Geiger SB, Levin L. Patients’ satisfaction with dental esthetics. J Am Dent Assoc. 2007;138:805-8.
29. Enabulele JE, Omo JO. Self perceived satisfaction with dental appearance and desired treatment to improve aesthetics. Afr J Oral Health. 2017;7:1-7.
30. El Mourad AM, Al Shamrani A, Al Mohaimeed M, Al Sougi S, Al Ghanem S, Al Manie W. Self-perception of dental esthetics among dental students at King Saud University and their desired treatment. Int J Dent. 2021;2021: 6671112.
31. Singh A, Peres MA, Watt RG: The relationship between income and oral health: a critical review. J Dent Res. 2019;98:853-60.
32. Manski RJ, Moeller JF, Chen H, St Clair PA, Schimmel J, Pepper JV. Wealth effect and dental care utilization in the United States. J Public Health Dent. 2012, 72:179-89.
33. AlSagob EI, Alkeait F, Alhaimy L, Alqahtani M, Hebbal M, Ben Gassem AA. Impact of self-perceived dental esthetic on psychosocial well-being and dental self-confidence: a cross-sectional study among female students in Riyadh city. Patient Prefer Adherence. 2021;15:919-26.
34. Alhajj MN, Ariffin Z, Celebić A, Alkheraif AA, Amran AG, Ismail IA. Perception of orofacial appearance among laypersons with diverse social and demographic status. PLoS One. 2020; 15: e0239232.
35. Manipal S, Mohan CS, Kumar DL, Cholan PK, Ahmed A, Adusumilli P. The importance of dental aesthetics among dental students assessment of knowledge. J Int Soc Prev Community Dent. 2014;4:48-51.
