# Supplementary material for: Dental aesthetic self-perception, smile satisfaction and associated factors among adults: A cross-sectional study
Source: PLoS One. 2026 Sep 15;21(9):e0357126. doi: 10.1371/journal.pone.0357126 (PMC13577522; doi:10.1371/journal.pone.0357126)
Supplement: Supplementary file 8 [file pone.0357126.s008.docx]

ONE-D-25-45043

Dental aesthetic self-perception, smile satisfaction and associated factors among adults: a cross-sectional study

PLOS ONE

**Journal Requirements:**

When submitting your revision, we need you to address these additional requirements.

1. Please ensure that your manuscript meets PLOS ONE's style requirements, including those for file naming. The PLOS ONE style templates can be found at https://journals.plos.org/plosone/s/file?id=wjVg/PLOSOne_formatting_sample_main_body.pdf and https://journals.plos.org/plosone/s/file?id=ba62/PLOSOne_formatting_sample_title_authors_affiliations.pdf

**Response:** We have checked, and our manuscript follows PLOS ONE's style requirements.

2. PLOS requires an ORCID iD for the corresponding author in Editorial Manager on papers submitted after December 6th, 2016. Please ensure that you have an ORCID iD and that it is validated in Editorial Manager. To do this, go to ‘Update my Information’ (in the upper left-hand corner of the main menu), and click on the Fetch/Validate link next to the ORCID field. This will take you to the ORCID site and allow you to create a new iD or authenticate a pre-existing iD in Editorial Manager.

**Response:** The corresponding author’s ORCID iD has been validated.

3. Please include a complete copy of PLOS’ questionnaire on inclusivity in global research in your revised manuscript. Our policy for research in this area aims to improve transparency in the reporting of research performed outside of researchers’ own country or community. The policy applies to researchers who have travelled to a different country to conduct research, research with Indigenous populations or their lands, and research on cultural artefacts. The questionnaire can also be requested at the journal’s discretion for any other submissions, even if these conditions are not met.  Please find more information on the policy and a link to download a blank copy of the questionnaire here: https://journals.plos.org/plosone/s/best-practices-in-research-reporting. Please upload a completed version of your questionnaire as Supporting Information when you resubmit your manuscript.

**Response:** The questionnaire has been attached as supporting information.

 4. We note that there is identifying data in the Supporting Information file <S1 File.csv>.

**Response:** All identifying information has been anonymized.

5. If the reviewer comments include a recommendation to cite specific previously published works, please review and evaluate these publications to determine whether they are relevant and should be cited. There is no requirement to cite these works unless the editor has indicated otherwise.

**Response:** Thanks for pointing this out.

Additional Editor Comments:

Major methodological corrections. See details in the Reviewers' reports.

**Response:** All reviewers’ suggestions and comments have been incorporated into the manuscript.


Reviewers' comments:

Reviewer's Responses to Questions

**Comments to the Author**

1. Is the manuscript technically sound, and do the data support the conclusions?

The manuscript must describe a technically sound piece of scientific research with data that supports the conclusions. Experiments must have been conducted rigorously, with appropriate controls, replication, and sample sizes. The conclusions must be drawn appropriately based on the data presented.

Reviewer #1: Yes

Reviewer #2: Yes

**Response:** Thank you for your comments.

2. Has the statistical analysis been performed appropriately and rigorously?

Reviewer #1: Yes

Reviewer #2: Yes

**Response:** Thank you for your comments.

3. Have the authors made all data underlying the findings in their manuscript fully available?

The [PLOS Data policy](http://www.plosone.org/static/policies.action#sharing) requires authors to make all data underlying the findings described in their manuscript fully available without restriction, with rare exception (please refer to the Data Availability Statement in the manuscript PDF file). The data should be provided as part of the manuscript or its supporting information, or deposited to a public repository. For example, in addition to summary statistics, the data points behind means, medians and variance measures should be available. If there are restrictions on publicly sharing data—e.g. participant privacy or use of data from a third party—those must be specified.

Reviewer #1: Yes

Reviewer #2: Yes

**Response:** Thank you for your comments.

4. Is the manuscript presented in an intelligible fashion and written in standard English?

PLOS ONE does not copyedit accepted manuscripts, so the language in submitted articles must be clear, correct, and unambiguous. Any typographical or grammatical errors should be corrected at revision, so please note any specific errors here.

Reviewer #1: No

Reviewer #2: Yes

**Response:** Thank you for your comments. The whole manuscript has been checked for spelling and grammar. General editing has been done throughout the manuscript. Additionally, we have used Grammarly to check the manuscript and improve sentence structures.

5. Review Comments to the Author

Please use the space provided to explain your answers to the questions above. You may also include additional comments for the author, including concerns about dual publication, research ethics, or publication ethics. (Please upload your review as an attachment if it exceeds 20,000 characters)

Reviewer #1: Dear authors
This very important article has a very important message.

**Response:** Thank you very much for liking the research idea.

This article could be published after following major corrections:
1. Title: Considering adding a pilot study since the sample can be much larger for Pakistan.

**Response:** Thank you for this valuable comment. We respectfully submit that the term “pilot study” may not be appropriate for this manuscript. Pilot studies are typically conducted to test feasibility, instruments, or procedures and are not primarily intended to generate substantive findings. In contrast, the present study was designed as a full cross-sectional investigation with a priori sample size calculation, a large final sample (n = 854), and inferential statistical analyses, including multivariable logistic regression.

Although Pakistan has a large population, the adequacy of a study sample is determined by study design, objectives, and statistical power rather than national population size alone. The final sample exceeded the minimum required sample size and provided sufficient power to address the study objectives.

We acknowledge the reviewer’s concern regarding generalizability. To address this, we have clearly stated in the **limitations section** that the study was conducted in a single metropolitan city using convenience sampling, which may limit national representativeness. We believe this clarification more accurately reflects the scope of the study than labeling it as a pilot.

2. Abstract: Methods: The online questionnaire link was distributed via email and social media apps. – Please specify to whom the questioners have been distributed. For example, the population of Karachi.
**Response:** Thank you for pointing this out. We have added this point to the manuscript. The questionnaire was distributed among the population of Karachi.

3. Abstract: Results: Eight hundred fifty-four individuals participated in the study, comprising 276 males (32.3%) and 578 females (67.7%). – Please specify to which population. Is it worldwide or just Pakistan? Is it Karachi?

**Response:** Thank you for pointing this out. We have added this point to the manuscript. This was the population of Karachi.

4. Introduction: A study conducted in Saudi Arabia in 2023 revealed that the most common dental issue affecting people's satisfaction was inadequate filling of front teeth [9]. – Please replace the word filling with restoration.
**Response:** Thank you for pointing this out. We have replaced the word filling with restoration.

5. Introduction: Multiple studies, including one by Nazir R in 2014 and another by Gassem AA in 2022, indicated that malocclusion, particularly in the front teeth, negatively impacts an individual's personality and self-esteem [10,11]. – Don’t use authors' names. Please remove authors' names and rewrite the sentence. Please correct the entire article.

**Response:** Thank you for pointing this out. We have removed the authors’ names.

6. Introduction: When malocclusion, especially in the front teeth, is present, it triggers negative social responses, ultimately leading to a reduced self-image [10,11]. – Please rewrite the sentence. Please change the front teeth to anterior teeth. For example, upper and lower anterior teeth.

**Response:** Thank you for pointing this out. We have replaced the word front with anterior and the sentence has been rephrased.

7. Discussion: A significant majority of the participants expressed admiration for another person's smile. This finding highlights the widespread recognition and appreciation of the positive effect of a warm and genuine smile on interpersonal interactions. – Please specify that this is a current study result.

**Response:** Thank you for pointing this out. We have modified the sentence to include the current study’s results.

8. Discussion: The results parallel those of a Saudi study – Please rewrite the sentence.

**Response:** Thank you for pointing this out. We have rewritten the sentence.

9. Discussion: Thirty-nine percent of participants reported frequently or occasionally avoiding smiling when their photographs were taken. Notably, there was no difference in opinion between the sexes. Participants provided various reasons for this behavior, including concerns regarding the appearance of their teeth in terms of size, shape, and color, as well as misalignments and gaps. – Please specify that this is a current study result. Please correct the word color.

**Response:** Thank you for pointing this out. We have modified the sentence to include the current study’s results. We have corrected the word color.

10. Discussion: Our research shows that most individuals surveyed agree on the significant influence a pleasant smile can have on a person's overall demeanor. – Don’t use personal language(our). Rather start the sentence with the current study. Please correct spelling for a word demeanor.

**Response:** Thank you for pointing this out. We have modified the sentence. We have corrected the spelling of the word demeanour.

11. Discussion: Please state the importance of the study. Please explain what value this study adds to already available information.

**Response:** Thank you for pointing this out. The following paragraph has been added to the manuscript.

**Importance of the study**

The study is important because it provides population-based data regarding the perception of adults in terms of their dental aesthetics and their satisfaction with their smiles and how these perceptions relate to their psychosocial well-being in an urban setting in South Asia. Most of the published studies on the topic are from Western or Middle Eastern backgrounds. The general adult population in Pakistan, and particularly in Karachi, has only limited data. Therefore, the current study fills this gap and provides culturally and regionally applicable information on the issue of smile perception and satisfaction in adults.

The research contributes to the current body of knowledge in a number of significant aspects. It emphasizes that over one-third of adults are unhappy with their smiles, but sociodemographic factors of age, sex, education, and occupation did not have a strong connection with smile satisfaction. It is important to note that the present study results defy the belief in the demographic factors of smile satisfaction, and highlight the predominance of the personal perceptions and subjective experience that is usually under-researched in dental public health studies. The present research supports and builds on previous research regarding the psychosocial significance of smiling, as it indicates that dental aesthetics may not be regarded as a cosmetic factor only, but rather as a part of a general QoL. The present study findings can be used to improve the relationship between clinical evaluation and patient expectations and assist in better patient-focused treatment planning and give priority to affordable aesthetic and orthodontic services in resource-constrained settings.

12. Implication of the study: Our results indicate several exciting findings. - Don’t use personal language(our).

**Response:** Thank you for pointing this out. We have modified the sentence.

13. Implication of the study: Our findings have significant implications for all stakeholders. - Don’t use personal language(our).

**Response:** Thank you for pointing this out. We have modified the sentence.

14. Conclusion: Please rewrite the conclusion.

**Response:** A few points in the conclusion section have been rewritten.

15. General comments: Discussion: Please add references.

**Response:** Thank you for pointing this out. Five new references have been added (four from 2025 and one from 2026 published studies).

16. General comments: Discussion: It is very important to highlight why this study is important and different as many others that are addressing similar or same topics. Is it only the Pakistani population? Is it Karachi?

**Response:** Thank you for pointing this out. A section on the importance of the present study and its value to the existing literature has been added to the discussion section.
17. General comments: Please correct grammar and spelling.

**Response:** Thank you for pointing this out. The whole manuscript has been checked for spelling and grammar. Additionally, we have used Grammarly to check the manuscript.
18. Please submit a similarity index.

**Response:** The similarity report is attached with the revised manuscript.

Reviewer #2: Title and Abstract:
• Title indicated study design
• Gives a brief about what the article is about, significance of the study while the aim is stated clearly.
**Response:** Thank you
Introduction :
• Line 77 and 78 remove Author initials

**Response:** Thank you for the suggestions. The authors’ names have been deleted.
• Literature review was not clear on the associated factors impacting the smile perception.

**Response:** Thank you for the comments. Please check line No 68-83. Several research articles have been mentioned regarding associated factors impacting the smile perception. Few more studies have been mentioned in the discussion section.

• Rationale clearly stated

**Response:** Thank you
• Aim clearly mentioned

**Response:** Thank you
• Objectives not stated, please mention them

**Response:** Thank you for pointing this out. We have rephrased the sentence “Consequently, this study was designed with the objectives to explore dental aesthetic self-perception, smile satisfaction, and related factors among a sample of the adult general population in Karachi, Pakistan.”

Methods:
• Study population not clearly described

**Response:** Thank you for the comments. The study population has been described in the revised manuscript. Please check lines No. 118-125.

• Study setting not explained

**Response:** Thank you for the comments. The study setting has been explained in the revised manuscript. Please check lines No. 102-109.


• Inclusion and exclusion criteria not clearly stated

**Response:** Thank you for the comments. The inclusion and exclusion criteria have been clearly stated in the revised manuscript. Please check lines No. 118-125.

• No clear description of how the individual variables were measured, where the study questionnaire was adapted from and was it validated??

**Response:** Thank you for the comments. The description of individual variables measurement have mentioned in the revised manuscript. Please check lines No. 127-30.
Two references for study questionnaire have already been given. Yes, it was validated.

“The researchers designed an online questionnaire and included a few questions from already published, well-known, validated questionnaires, namely, the "Aesthetic Component (AC) of the Index of Orthodontic Treatment Need (IOTN)" and the "Psychosocial Impact of Dental Aesthetics Questionnaire (PIDAQ)" [17,18]. Two subject experts checked the content and construct validity of the questionnaire, and modifications were made according to their suggestions. A pilot survey was conducted with 50 individuals to assess questionnaire understanding and readability. After the pilot study, the questionnaire was further modified. The construction of the present questionnaire, including the use of questions from well-tested scales (IOTN-AC and PIDAQ), expert review, and pilot testing, supports its face and content validity.”

Results:
• What was the response rate and explain why

**Response:** Thank you for the comments.

This study used an online questionnaire distributed through email and multiple social media platforms (WhatsApp, Facebook, Instagram, and Twitter) using a convenience sampling approach. The survey link was shared broadly through personal networks, friends, and family contacts, and participants were encouraged to forward it further. As a result, it was not possible to determine the exact number of individuals who received or viewed the survey invitation.

Because the denominator (total number of people invited) was unknown, a conventional response rate (number of completed questionnaires divided by number of individuals approached) could not be computed. This is a common and accepted limitation of online, open-link, convenience-sampled surveys, particularly those conducted via social media platforms.

• There is inconsistency on the mentioned sample size of 386 and the result mention of a total of 864 participants

**Response:** Thank you for the comments.

The figure of **386 participants** refers to the **minimum required sample size**, which was calculated **prior to data collection** using the Raosoft sample size calculator. This calculation was based on standard assumptions (95% confidence level, 5% margin of error, and an anticipated response distribution of 50%) to ensure adequate statistical power for the study.

However, during the data collection period, the online questionnaire continued to receive responses beyond the minimum required number. As a result, a **larger number of participants completed the survey**, and all eligible and complete responses were included in the final analysis. Ultimately, **854 participants** met the inclusion criteria and were analyzed.

Including a sample size larger than the minimum calculated requirement is methodologically acceptable and, in fact, **strengthens the study**, as it:

- Improves the precision of estimates,
- Increases statistical power, and
- Enhances the representativeness of the findings within the target population.

The difference between the **calculated sample size (386)** and the **final analyzed sample (854),** therefore, reflects **over-recruitment**, not an error in methodology.

• On the tables, use small letter n, for the study proportions and a small letter p for p value

**Response:** Thank you for the comments. In all the tables, small letter n, for the study proportions, and a small letter p for p-value have written.

• General editing required

**Response:** Thank you for pointing this out. The whole manuscript has been checked for spelling and grammar. General editing has been done throughout the manuscript. Additionally, we have used Grammarly to check the manuscript.

• No clear mention of the reference group in the regression analysis
**Response:** Thank you for pointing this out. In variable age (18-20 was the reference group), gender (female was the reference group), marital status (Divorced was the reference group), education (Graduate was the reference group) and occupation (Government job was the reference group). In front of each reference group, it has been mentioned.

Discussion:

• Some statements need references, e.g. line 216 and 218

**Response:** Thank you for pointing this out. We are sorry for this mistake. The appropriate references have been cited.

• Sample size mentioned in the limitations, explain the limitation and mention how to overcome it.

**Response:** Thank you for pointing this out. This has been mentioned in the revised version.

• Statement on line 259, please explain. Did you control for confounding in the regression analysis??

**Response:** Thank you for pointing this out.

In line 259 it has been mentioned that “Several confounding factors that we were unable to examine have been emphasized in the literature as having a significant impact on self-perceived dental aesthetics and psychosocial well-being.”

We have explained those factors provided references in the subsequent paragraph. “One study indicated that university affiliation (private vs. public), study discipline, college, and income levels were all significant factors affecting these perceptions [37]. Another study from Saudi Arabia found that “urban residence, tooth color, crowding, unsatisfactory frontal teeth fillings, broken frontal teeth, red inflamed gums, covering teeth while smiling, and the degree of gum display while smiling” were associated with satisfaction regarding dental aesthetics [9]. Furthermore, a study noted that among laypersons, a positive perception of orofacial appearance was related to general health conditions, higher educational levels, and perceptions of oral health conditions [38].”

Regarding confounding factors, in this study, potential confounding factors were partially controlled by multivariable binary logistic regression with the “Enter method”. By entering all sociodemographic variables, such as gender, age, marital status, and education, into the model, the analysis estimated the independent association of each predictor with smile satisfaction while adjusting for the influence of the other variables. This approach reduces confounding by ensuring that the observed effect of any single variable reflects its association with smile satisfaction net of other measured factors included in the model.

General comments:
• Good study with important findings

**Response:** Thank you for your comments.

• The article needs some editing.

**Response:** Thank you for pointing this out. The whole manuscript has been checked for spelling and grammar. General editing has been done throughout the manuscript. Additionally, we have used Grammarly to check the manuscript.

6. PLOS authors have the option to publish the peer review history of their article ([what does this mean?](https://journals.plos.org/plosone/s/editorial-and-peer-review-process#loc-peer-review-history)). If published, this will include your full peer review and any attached files.


If you choose “no”, your identity will remain anonymous but your review may still be made public.


**Do you want your identity to be public for this peer review?** For information about this choice, including consent withdrawal, please see our [Privacy Policy](https://www.plos.org/privacy-policy).

Reviewer #1: **Yes:** Prof Vesna Karic

Reviewer #2: **Yes:** Dr CB MALAU
