# Supplementary material for: Dental aesthetic self-perception, smile satisfaction and associated factors among adults: A cross-sectional study
Source: PLoS One. 2026 Sep 15;21(9):e0357126. doi: 10.1371/journal.pone.0357126 (PMC13577522; doi:10.1371/journal.pone.0357126)
Supplement: Supplementary file 9 [file pone.0357126.s010.docx]

PONE-D-25-45043R1

Dental aesthetic self-perception, smile satisfaction and associated factors among adults: a cross-sectional study

PLOS One

Reviewers' comments:
Reviewer's Responses to Questions

**Comments to the Author**

1. If the authors have adequately addressed your comments raised in a previous round of review and you feel that this manuscript is now acceptable for publication, you may indicate that here to bypass the “Comments to the Author” section, enter your conflict of interest statement in the “Confidential to Editor” section, and submit your "Accept" recommendation.

Reviewer #1: All comments have been addressed

**Response:** Thank you

Reviewer #3: (No Response)

**Response:**

2. Is the manuscript technically sound, and do the data support the conclusions?
The manuscript must describe a technically sound piece of scientific research with data that supports the conclusions. Experiments must have been conducted rigorously, with appropriate controls, replication, and sample sizes. The conclusions must be drawn appropriately based on the data presented.

Reviewer #1: Yes

**Response:** Thank you

Reviewer #3: Partly

**Response:** We have modified the manuscript as suggested. Hopefully, now the manuscript is technically sound.

3. Has the statistical analysis been performed appropriately and rigorously?

Reviewer #1: Yes

**Response:** Thank you

Reviewer #3: I Don't Know

4. Have the authors made all data underlying the findings in their manuscript fully available?

Reviewer #1: Yes

**Response:** Thank you

Reviewer #3: No

**Response:** We have uploaded the CSV file of the data. So all the data given in the manuscript is freely available.

5. Is the manuscript presented in an intelligible fashion and written in standard English?

PLOS ONE does not copyedit accepted manuscripts, so the language in submitted articles must be clear, correct, and unambiguous. Any typographical or grammatical errors should be corrected at revision, so please note any specific errors here.

Reviewer #1: Yes

**Response:** Thank you

Reviewer #3: Yes

**Response:** Thank you

6. Review Comments to the Author

Please use the space provided to explain your answers to the questions above. You may also include additional comments for the author, including concerns about dual publication, research ethics, or publication ethics. (Please upload your review as an attachment if it exceeds 20,000 characters)

**Reviewer #1:** (No Response)

**Reviewer #3:** The manuscript lacks essential and basic information that is standard in a scientific piece. The interpretation of the results and discussions thereof is not convincing, and the conclusion does not tie to the evidence presented. The authors should look at explaining why, how and when: answering these questions may help fill in the gaps.
Lastly formatting will be necessary, and all tables & Figures must be on the same page as the description.
All comments are made in the text in the manuscript.

**Response:** Thank you for comments. We appreciate reviewer’s keen observation and constructive comments. We have modified the manuscript and addressed all points suggested by the reviewer. Sometimes, it is difficult to keep figure or table on the same page where description is given because of the continuity of the text or length of the table.

Please add references and percentages of how much each one was reported to be prominent.

**Response:** The following reference and percentages have been added.

A study reported that the eyes contribute 25%, the mouth 18%, face shape 15%, Skin 12%, and the nose 6% to the overall assessment of facial attractiveness (Ibrahim et al., 2024).

If the use of adolescent here is meant to indicate low...rather refer toit as low self esteem.

**Response:** Thank you for the suggestion. We have corrected it.

Is this limited to adults only? Do children and young adults not suffer the same fate?

**Response:** You are right. Thank you for pointing this out. We have rephrased it. Actually, we have used social interactions, quality of life, which is why we used adults.

Except in this study participants will be assessing their own smiles make this statement relevant to the discussion.

**Response:** Thank you for the suggestion. We have shifted this statement from the introduction to discussion section.

What is filling referring to here: restorations OR space-diastemata?

**Response:** Here, filling is referring to gap or space between two or more teeth,

Please provide a reference for this statement.

**Response:** The following references have been written. The reference numbers 10 and 11.

“The way individuals perceive their appearance significantly influences their personality and can trigger negative social responses that may ultimately lead to a reduced self-image [10,11].”

This will also help manage patients, expectations.

**Response:** Thank for the suggestions. We have incorporated this phrase into the sentence.

Please name the related factors.

**Response:** The names of the related factors have been included.

This study aimed to explore dental aesthetic self-perception, smile satisfaction, and related sociodemographic factors, including age, gender, marital status, education, and occupation, among a sample of the general adult population in Karachi, Pakistan.

State clearly who were the participants and how they were chosen &/or contacted to participate on the online survey.

**Response:** The study participants were recruited from the general population of Karachi via an online questionnaire. The questionnaire link was distributed via email and social media platforms, including WhatsApp, Facebook, Instagram, and Twitter, and two reminders were also sent at one-week intervals. Additionally, the researchers used their classmates, friends, and family contacts for recruiting study participants.

Were the participants not completing the survey? How long were they given?

**Response:** The researchers used their classmates, friends, and family contacts to recruit more study participants. The questionnaire link was distributed via email and social media platforms, including WhatsApp, Facebook, Instagram, and Twitter, and two reminders were also sent at one-week intervals.

Is this urban/ rural/ peri-urban?

**Response:** Karachi is a cosmopolitan city and the most developed urban area in Pakistan. This sentence has been included in the method section.

How was this sample size determined?

**Response:** The sample size determination method has been included in the sentence.

The sample size, 386, was determined before the start of the study using the Raosoft sample size calculator (<http://www.raosoft.com/samplesize.html>. Accessed on October 20, 2022) with a 5% margin of error and a 95% confidence level, using a population of 20 million for Karachi. The sample size was increased to enhance the survey's generalizability.

When was this accessed: web address & date?

**Response:** The web address & date have been included in the sentence.

The sample size, 386, was determined before the start of the study using the Raosoft sample size calculator (<http://www.raosoft.com/samplesize.html>. Accessed on October 20, 2022) with a 5% margin of error and a 95% confidence level, using a population of 20 million for Karachi. The sample size was increased to enhance the survey's generalizability. Generally, the larger sample improves the survey's precision and reliability and enables better subgroup analyses.

How were these identified...what made them relevant for your study?

**Response:** Two subject-matter experts assessed the content and construct validity of the questionnaire, and modifications were made based on their suggestions.

Please cite the software correctly and in full.

**Response:** Data were analyzed using **IBM** Statistical Package for the Social Sciences (**SPSS) for Windows, Version 26.0** (IBM Corp., Armonk, NY, USA).

When did it become this and not 386?

**Response:** Thank you for this important comment. The calculated sample size of 384 represented the **minimum required sample** for adequate statistical precision. We collected 854 responses because larger sample sizes are acceptable and can strengthen survey-based studies. The increased sample improved the precision and reliability of the estimates, reduced sampling error, and allowed for better subgroup analyses. In addition, questionnaire-based studies often anticipate incomplete or invalid responses; therefore, collecting more responses helps ensure sufficient analyzable data. Since eligible responses continued to be received during the study period, all complete responses were included to enhance the representativeness and robustness of the findings.

Could not find figure 1.

**Response:** If figures are missing, we apologize. Actually, both figures were uploaded separately as required by the journal. Usually, these are present at the end of the PDF file that’s sent to the reviewer.

Could not locate figure 2

**Response:** If figures are missing, we apologize. Actually, both figures were uploaded separately as required by the journal. Usually, these are present at the end of the PDF file that’s sent to the reviewer.

Please show the different findings from the various studies in percentages within text

**Response: Two dissimilar references have been added.**

Please add percentages from each study in-text.

**Response:** The percentages have been added.

Please add relevant percentages.

**Response:** The relevant percentages have been added.

References please.

**Response:** The references have been added.

Please quantify the ‘significant majority’.

**Response:** The percentage has been added to quantify the ‘significant majority’.

Is it genuine smile OR being able to smile?

**Response:** The sentence has been modified by removing word ‘genuine’.

Observed between genders, or age-groups, or level of education?

**Response: Actually, this is the overall agreement not** between genders, or age-groups, or level of education

Overall level of agreement (85.9%) observed in this study regarding the impact of tooth color on smile attractiveness emphasizes the significance of tooth appearance.

State the percentage please

**Response:** The percentage has been added.

318(37,2%) versus 62,8%

**Response:** The percentage has been added

 To ensure your figures meet our technical requirements, please review our figure guidelines: <https://journals.plos.org/plosone/s/figures>

**Response:** Yes figure meet the journal’s technical requirements

You may also use PLOS’s free figure tool, NAAS, to help you prepare publication quality figures: <https://journals.plos.org/plosone/s/figures#loc-tools-for-figure-preparation>.

NAAS will assess whether your figures meet our technical requirements by comparing each figure against our figure specifications.

**Response:** Yes, We have used NAAS for preparing ou figure meet the journal’s technical requirements
