# Supplementary material for: Dental aesthetic self-perception, smile satisfaction and associated factors among adults: A cross-sectional study
Source: PLoS One. 2026 Sep 15;21(9):e0357126. doi: 10.1371/journal.pone.0357126 (PMC13577522; doi:10.1371/journal.pone.0357126)
Supplement: Supplementary file 10 [file pone.0357126.s011.docx]

**Dear Editor and Reviewers,**

We sincerely thank the editor and reviewers for their careful evaluation and constructive comments. We have revised the manuscript accordingly. All modifications are shown using the tracked changes format. In addition to the requested revisions, we corrected several internal inconsistencies and language problems to improve accuracy, transparency, and readability.

Sincerely,

Dr Javaria Baig

Journal Requirements:

If the reviewer comments include a recommendation to cite specific previously published works, please review and evaluate these publications to determine whether they are relevant and should be cited. There is no requirement to cite these works unless the editor has indicated otherwise. 

Please review your reference list to ensure that it is complete and correct. If you have cited papers that have been retracted, please include the rationale for doing so in the manuscript text, or remove these references and replace them with relevant current references. Any changes to the reference list should be mentioned in the rebuttal letter that accompanies your revised manuscript. If you need to cite a retracted article, indicate the article’s retracted status in the References list and also include a citation and full reference for the retraction notice.

**Response:** The reference list was reviewed for consistency with the citations used in the manuscript. No reviewer-requested reference was added because no specific publication was recommended. No change to the reference list was required for the present revision. The authors should nevertheless complete the journal’s final database-based retraction check during submission.

A dedicated Data availability statement has been added to make explicit that all data underlying the findings are supplied as S1 File.

**Reviewers' comments:**
**Comments to the Author**

1. If the authors have adequately addressed your comments raised in a previous round of review and you feel that this manuscript is now acceptable for publication, you may indicate that here to bypass the “Comments to the Author” section, enter your conflict of interest statement in the “Confidential to Editor” section, and submit your "Accept" recommendation.

Reviewer #1: All comments have been addressed

Reviewer #3: All comments have been addressed

Reviewer #4: (No Response)

2. Is the manuscript technically sound, and do the data support the conclusions?

The manuscript must describe a technically sound piece of scientific research with data that supports the conclusions. Experiments must have been conducted rigorously, with appropriate controls, replication, and sample sizes. The conclusions must be drawn appropriately based on the data presented.

Reviewer #1: Yes

Reviewer #3: Yes

Reviewer #4: Partly

3. Has the statistical analysis been performed appropriately and rigorously?

Reviewer #1: Yes

Reviewer #3: I Don't Know

Reviewer #4: N/A

4. Have the authors made all data underlying the findings in their manuscript fully available?

The [PLOS Data policy](http://track.editorialmanager.com/CL0/http:%2F%2Fwww.plosone.org%2Fstatic%2Fpolicies.action%23sharing/1/010f019f7d16e518-4042dc8a-e050-47ff-a492-2f9346421972-000000/eLaH8wYAt8n8--nQv7YBj3rTq2f63aBwtZy2Bqy24zc=258) requires authors to make all data underlying the findings described in their manuscript fully available without restriction, with rare exception (please refer to the Data Availability Statement in the manuscript PDF file). The data should be provided as part of the manuscript or its supporting information, or deposited to a public repository. For example, in addition to summary statistics, the data points behind means, medians and variance measures should be available. If there are restrictions on publicly sharing data—e.g. participant privacy or use of data from a third party—those must be specified.

Reviewer #1: Yes

Reviewer #3: Yes

Reviewer #4: No

5. Is the manuscript presented in an intelligible fashion and written in standard English?

PLOS ONE does not copyedit accepted manuscripts, so the language in submitted articles must be clear, correct, and unambiguous. Any typographical or grammatical errors should be corrected at revision, so please note any specific errors here.

Reviewer #1: Yes

Reviewer #3: Yes

Reviewer #4: Yes

6. Review Comments to the Author

Please use the space provided to explain your answers to the questions above. You may also include additional comments for the author, including concerns about dual publication, research ethics, or publication ethics. (Please upload your review as an attachment if it exceeds 20,000 characters)

**Reviewer 1**

Congratulations on your article. Please correct following before publication:
1. Malocclusion, particularly in the front teeth, negatively impacts an individual's personality and self-esteem [10,11], - Add full stop.

**Response:** Thank you. We corrected the punctuation and also revised the surrounding sentences for grammar and clarity.

# **Reviewer 3**

We thank Reviewer 3 for confirming that the previous comments were adequately addressed and for indicating that the manuscript is technically sound and clearly written. No additional revision was requested.

**Reviewer 4**

1. SUMMARY OF THE MANUSCRIPT
This cross-sectional study investigated dental aesthetic self-perception, smile satisfaction, and associated psychosocial factors in 854 adults recruited from Karachi, Pakistan. Using a self-administered online questionnaire, distributed via email and social media between November 2022 and March 2023. The authors report that 37.2% of participants were dissatisfied with their smiles, that the majority of the participants linked smiling to mental and social well-being, and that there was no significant associations found between sociodemographic variables and smile satisfaction in binary logistic regression.

**Response:** Thank you

2. STRENGTHS
• The sample size was adequate (n=854)
• The study addresses clinically and psychologically topics that are relevant, which then will mean that dental care will be patient-centred in their country’s context.
• The research question is clearly articulated and the study fills a real gap in the published literature on smile satisfaction in Pakistan population
• Inclusion of gender-disaggregated analysis provides useful insight into different perceptions of smile aesthetics

**Response:** Thank you

3. MAJOR CONCERNS
3.1 Sampling Strategy and Selection Bias
With the study relying on convenience sampling via social media and email, it introduces an element of selection bias. Only those with better digital literacy, and stronger health awareness are overrepresented in online surveys. This will be problematic in a city like Karachi, which has a diverse socio-economic landscape, with the lager population group may lack consistent access to internet.

**Response:** We agree. The method section now identifies the recruitment strategy explicitly as online convenience sampling. The limitations section has been substantially expanded to explain likely overrepresentation of younger, educated, digitally literate, health-aware, and internet-connected participants; underrepresentation of people with limited digital access; use of personal networks; single-city recruitment; and the fact that a large sample cannot remove selection bias.

3.2 Absence of a Validated Instrument
The manuscript does not state whether the questionnaire was validated or adapted from an established, validated instrument. Dental aesthetics self-perception research commonly use validated tools such as the Oral Aesthetics Subjective Impact Scale (OASIS)
**Response:** Thank you for identifying the need for clearer reporting. The study used a researcher-developed questionnaire incorporating selected questions and concepts from the IOTN Aesthetic Component and PIDAQ, rather than administering either complete instrument. The questionnaire was pilot-tested in 50 individuals and reviewed by two subject-matter experts for content relevance and clarity. The available validation evidence was limited to pilot testing and expert review; reliability coefficients and factor-analytic validation were not conducted. We now state this transparently in both the methods and limitations. OASIS was not used in this study; therefore, we have not presented the instrument as OASIS or added it as the source of the questionnaire.

3.3 Statistical Reporting Errors
Several statistical inconsistencies are present in the abstract and results:
• The p-value for gender analysis of tooth colour is reported as “p<0.034” This is non-standard. If the p value is already below 0.05 then the correct reporting format should be “p=0.034”
• Rounding of percentages is not consistent: the author rounded of 69.67% to 69.7% (to those agreed that smile can impact mental , emotional and social wellbeing. But then rounded off 77,87% to 77,95% which the mumber are not the same
**Response:** We agree. The exact p-value is now reported as p = 0.034, and 665/854 is consistently reported as 77.9%. We also standardized p-value spacing and percentage presentation. During this check, we found that the narrative statements claiming consistently higher endorsement among females did not accurately reflect the category percentages in Table 3. We therefore revised the text to report significant overall gender differences in response distributions without assigning an unsupported direction.

3.4 No mention of Ethical Approval and Informed Consent Documentation
There is no mention of ethical approval from the relevant board or Ethics Committee, nor is there any mention of informed consent procedures for online participation

**Response:** Ethical approval and consent information were present in the submitted Methods, but we agree that the procedure could be stated more explicitly. We have strengthened the description by retaining the LCMD Institutional Review Board reference number (IRB/D-000046/22) and explaining the pre-questionnaire information and consent statement, voluntary participation, electronic informed consent through proceeding and submission, anonymity, and use of the data for research publication.

4. MINOR CONCERNS
4.1 Introduction
The introduction as written in the abstract does not contextualise the study within the existing literature. No reference to any study in relation to smile satisfaction or dental aesthetics
**Response:** We agree. A concise contextual sentence has been added to the abstract, stating that dental aesthetic self-perception can influence self-confidence and psychosocial well-being and that evidence from Pakistan remains limited. The abstract objective has also been made more specific.

4.2 Methods and Results
• Inclusion and Exclusion criteria are not described

**Response:** The criteria were present in the original Methods but were embedded within the recruitment paragraph. We have reorganized and clarified them. Eligible participants were adults aged 18 years or older, male or female, and residents of Karachi. Exclusion criteria were previous orthodontic treatment and dental or facial injuries, conditions, or craniofacial anomalies such as cleft lip or palate.

• The gender breakdown is notable way to skew, 32.3% males, 67,7% females

**Response:** We agree. The unequal gender distribution is now explicitly acknowledged as a limitation affecting gender comparisons and generalizability. We also removed overinterpretation of gender differences from the results, discussion, and implications sections.

4.3 Discussion And Conclusion
• The claim that more that 50% of subjects expressed contentment with their smiles is inconsistence with the finding that 37.2% were dissatisfied.

**Response:** The two values are complementary: 536/854 participants (62.8%) were satisfied and 318/854 (37.2%) were dissatisfied. To avoid ambiguity, we replaced the phrase “more than 50%” with the exact value, 62.8%, in the abstract and conclusion.

• The discussion should compare findings with similar studies from South of Asia, Middle East and other comparable lower-middle income country context

**Response:** We agree. The comparative discussion has been consolidated and expanded using studies already cited in the manuscript. The revised text compares the present 62.8% satisfaction estimate with findings from Saudi Arabia, Vietnam, Malaysia, and Serbia, and additionally notes evidence from Pakistan and northern India regarding the psychosocial relevance of dental aesthetics and perceived orthodontic treatment need. Differences in age, culture, populations, sampling methods, and instruments are discussed as possible explanations for variation.

5. REQUIRED ACTIONS AD RECOMMENDATIONS
a) Provide IRB/Ethics Committee approval number and informed consent protocol details
**Response:** Provided

b) Validate or cite the validation status of the Questionnaire used
**Response:** Validation explained

c) Correct the p-value reporting error (p,0.034 …..p=0.034 and the percentage rounding off)
**Response:** all p-values have been corrected.

d) State the inclusion/exclusion criteria clearly in the Methods section
**Response:** Inclusion exclusion criteria has been explained.

e) Provide a sample size justification or power calculation
**Response:** sample size justification provided.

f) Discuss the selection criteria bias from internet -based convenience sampling explicitly in the Limitations section

**Response:** Selection bias has been mentioned in the limitations.

# **Additional accuracy and language corrections**

- Corrected the female percentage for noticing the nose first from 3.6% to 6.2% because 36/578 = 6.2%; the frequency count was unchanged.
- Corrected typographical and grammatical errors throughout the abstract, Methods, Results, and Discussion.
- Replaced imprecise or unsupported interpretations with wording that remains within the cross-sectional, self-reported data.
- Standardized terminology, including 'buccal corridor,' p-value formatting, and the presentation of frequencies and percentages.

7. PLOS authors have the option to publish the peer review history of their article ([what does this mean?](https://track.editorialmanager.com/CL0/https:%2F%2Fjournals.plos.org%2Fplosone%2Fs%2Feditorial-and-peer-review-process%23loc-peer-review-history/1/010f019f7d16e518-4042dc8a-e050-47ff-a492-2f9346421972-000000/ndr99S9-m44_UVKCE3llJQ52ty023FQi83G9Gjohq3I=258)). If published, this will include your full peer review and any attached files.


If you choose “no”, your identity will remain anonymous but your review may still be made public.


**Do you want your identity to be public for this peer review?** For information about this choice, including consent withdrawal, please see our [Privacy Policy](https://track.editorialmanager.com/CL0/https:%2F%2Fwww.plos.org%2Fprivacy-policy/1/010f019f7d16e518-4042dc8a-e050-47ff-a492-2f9346421972-000000/m14mYtLHeb7DeLUBpA_mBGfOYA24buqzkKsiKbOj2hg=258).

Reviewer #1: **Yes:** Prof Vesna Karic

Reviewer #3: No

Reviewer #4: **Yes:** Kinder Modisha

[NOTE: If reviewer comments were submitted as an attachment file, they will be attached to this email and accessible via the submission site. Please log into your account, locate the manuscript record, and check for the action link "View Attachments". If this link does not appear, there are no attachment files.]

To ensure your figures meet our technical requirements, please review our figure guidelines: <https://journals.plos.org/plosone/s/figures>

You may also use PLOS’s free figure tool, NAAS, to help you prepare publication quality figures: <https://journals.plos.org/plosone/s/figures#loc-tools-for-figure-preparation>.

NAAS will assess whether your figures meet our technical requirements by comparing each figure against our figure specifications.
