# Supplementary material for: Dental aesthetic self-perception, smile satisfaction and associated factors among adults: A cross-sectional study
Source: PLoS One. 2026 Sep 15;21(9):e0357126. doi: 10.1371/journal.pone.0357126 (PMC13577522; doi:10.1371/journal.pone.0357126)
Supplement: Peer review history

# Author comment: Author response to Decision Letter 0

**DOI:** 10.1371/journal.pone.0357126.r001

21 Aug 2025

Attachment Submitted filename: Rebuttal letter .docx

# Peer review: Decision Letter 0

**DOI:** 10.1371/journal.pone.0357126.r002

**Authors:** Yolanda Malele-Kolisa

9 Dec 2025

Dear Dr. Baig,

Thank you for submitting your manuscript to PLOS ONE. After careful consideration, we feel that it has merit but does not fully meet PLOS ONE’s publication criteria as it currently stands. Therefore, we invite you to submit a revised version of the manuscript that addresses the points raised during the review process.

Please submit your revised manuscript by Jan 23 2026 11:59PM. If you will need more time than this to complete your revisions, please reply to this message or contact the journal office at plosone@plos.org. When you're ready to submit your revision, log on to https://www.editorialmanager.com/pone/ and select the 'Submissions Needing Revision' folder to locate your manuscript file.

- A rebuttal letter that responds to each point raised by the academic editor and reviewer(s). You should upload this letter as a separate file labeled 'Response to Reviewers'.
- A marked-up copy of your manuscript that highlights changes made to the original version. You should upload this as a separate file labeled 'Revised Manuscript with Track Changes'.
- An unmarked version of your revised paper without tracked changes. You should upload this as a separate file labeled 'Manuscript'.

If you would like to make changes to your financial disclosure, please include your updated statement in your cover letter. Guidelines for resubmitting your figure files are available below the reviewer comments at the end of this letter.

If applicable, we recommend that you deposit your laboratory protocols in protocols.io to enhance the reproducibility of your results. Protocols.io assigns your protocol its own identifier (DOI) so that it can be cited independently in the future. For instructions see: https://journals.plos.org/plosone/s/submission-guidelines#loc-laboratory-protocols. Additionally, PLOS ONE offers an option for publishing peer-reviewed Lab Protocol articles, which describe protocols hosted on protocols.io. Read more information on sharing protocols at https://plos.org/protocols?utm_medium=editorial-email&utm_source=authorletters&utm_campaign=protocols.

We look forward to receiving your revised manuscript.

Kind regards,

Yolanda Malele-Kolisa, BDS, MPH, MDent, PhD

Academic Editor

PLOS ONE

Journal Requirements:

When submitting your revision, we need you to address these additional requirements.

1. Please ensure that your manuscript meets PLOS ONE's style requirements, including those for file naming. The PLOS ONE style templates can be found at https://journals.plos.org/plosone/s/file?id=wjVg/PLOSOne_formatting_sample_main_body.pdf and https://journals.plos.org/plosone/s/file?id=ba62/PLOSOne_formatting_sample_title_authors_affiliations.pdf

2. PLOS requires an ORCID iD for the corresponding author in Editorial Manager on papers submitted after December 6th, 2016. Please ensure that you have an ORCID iD and that it is validated in Editorial Manager. To do this, go to ‘Update my Information’ (in the upper left-hand corner of the main menu), and click on the Fetch/Validate link next to the ORCID field. This will take you to the ORCID site and allow you to create a new iD or authenticate a pre-existing iD in Editorial Manager.

3. Please include a complete copy of PLOS’ questionnaire on inclusivity in global research in your revised manuscript. Our policy for research in this area aims to improve transparency in the reporting of research performed outside of researchers’ own country or community. The policy applies to researchers who have travelled to a different country to conduct research, research with Indigenous populations or their lands, and research on cultural artefacts. The questionnaire can also be requested at the journal’s discretion for any other submissions, even if these conditions are not met.  Please find more information on the policy and a link to download a blank copy of the questionnaire here: https://journals.plos.org/plosone/s/best-practices-in-research-reporting. Please upload a completed version of your questionnaire as Supporting Information when you resubmit your manuscript.

4. We note that there is identifying data in the Supporting Information file <S1 File.csv>. Due to the inclusion of these potentially identifying data, we have removed this file from your file inventory. Prior to sharing human research participant data, authors should consult with an ethics committee to ensure data are shared in accordance with participant consent and all applicable local laws.

Data sharing should never compromise participant privacy. It is therefore not appropriate to publicly share personally identifiable data on human research participants. The following are examples of data that should not be shared:

-Name, initials, physical address

-Ages more specific than whole numbers

-Internet protocol (IP) address

-Specific dates (birth dates, death dates, examination dates, etc.)

-Contact information such as phone number or email address

-Location data

-ID numbers that seem specific (long numbers, include initials, titled “Hospital ID”) rather than random (small numbers in numerical order)

Data that are not directly identifying may also be inappropriate to share, as in combination they can become identifying. For example, data collected from a small group of participants, vulnerable populations, or private groups should not be shared if they involve indirect identifiers (such as sex, ethnicity, location, etc.) that may risk the identification of study participants.

Additional guidance on preparing raw data for publication can be found in our Data Policy (https://journals.plos.org/plosone/s/data-availability#loc-human-research-participant-data-and-other-sensitive-data) and in the following article: http://www.bmj.com/content/340/bmj.c181.long.

Please remove or anonymize all personal information (<specific identifying information in file to be removed>), ensure that the data shared are in accordance with participant consent, and re-upload a fully anonymized data set. Please note that spreadsheet columns with personal information must be removed and not hidden as all hidden columns will appear in the published file.

5. If the reviewer comments include a recommendation to cite specific previously published works, please review and evaluate these publications to determine whether they are relevant and should be cited. There is no requirement to cite these works unless the editor has indicated otherwise.

Additional Editor Comments :

Major methodological corrections. See details in the Reviewers' reports.

[Note: HTML markup is below. Please do not edit.]

Reviewers' comments:

Reviewer's Responses to Questions

**Comments to the Author**

1. Is the manuscript technically sound, and do the data support the conclusions?

Reviewer #1: Yes

Reviewer #2: Yes

**********

2. Has the statistical analysis been performed appropriately and rigorously?

Reviewer #1: Yes

Reviewer #2: Yes

**********

3. Have the authors made all data underlying the findings in their manuscript fully available?

The PLOS Data policy

Reviewer #1: Yes

Reviewer #2: Yes

**********

4. Is the manuscript presented in an intelligible fashion and written in standard English?

Reviewer #1: No

Reviewer #2: Yes

**********

Reviewer #1: Dear authors

This very important article has a very important message. This article could be published after following major corrections:

1. Title: Considering adding a pilot study since the sample can be much larger for Pakistan.

2. Abstract: Methods: The online questionnaire link was distributed via email and social media apps. – Please specify to whom the questioners have been distributed. For example, the population of Karachi.

3. Abstract: Results: Eight hundred fifty-four individuals participated in the study, comprising 276 males (32.3%) and 578 females (67.7%). – Please specify to which population. Is it worldwide or just Pakistan? Is it Karachi?

4. Introduction: A study conducted in Saudi Arabia in 2023 revealed that the most common dental issue affecting people's satisfaction was inadequate filling of front teeth [9]. – Please replace the word filling with restoration.

5. Introduction: Multiple studies, including one by Nazir R in 2014 and another by Gassem AA in 2022, indicated that malocclusion, particularly in the front teeth, negatively impacts an individual's personality and self-esteem [10,11]. – Don’t use authors' names. Please remove authors' names and rewrite the sentence. Please correct the entire article.

6. Introduction: When malocclusion, especially in the front teeth, is present, it triggers negative social responses, ultimately leading to a reduced self-image [10,11]. – Please rewrite the sentence. Please change the front teeth to anterior teeth. For example, upper and lower anterior teeth.

7. Discussion: A significant majority of the participants expressed admiration for another person's smile. This finding highlights the widespread recognition and appreciation of the positive effect of a warm and genuine smile on interpersonal interactions. – Please specify that this is a current study result.

8. Discussion: The results parallel those of a Saudi study – Please rewrite the sentence.

9. Discussion: Thirty-nine percent of participants reported frequently or occasionally avoiding smiling when their photographs were taken. Notably, there was no difference in opinion between the sexes. Participants provided various reasons for this behavior, including concerns regarding the appearance of their teeth in terms of size, shape, and color, as well as misalignments and gaps. – Please specify that this is a current study result. Please correct the word color.

10. Discussion: Our research shows that most individuals surveyed agree on the significant influence a pleasant smile can have on a person's overall demeanor. – Don’t use personal language(our). Rather start the sentence with the current study. Please correct spelling for a word demeanor.

11. Discussion: Please state the importance of the study. Please explain what value this study adds to already available information.

12. Implication of the study: Our results indicate several exciting findings. - Don’t use personal language(our).

13. Implication of the study: Our findings have significant implications for all stakeholders. - Don’t use personal language(our).

14. Conclusion: Please rewrite the conclusion.

15. General comments: Discussion: Please add references.

16. General comments: Discussion: It is very important to highlight why this study is important and different as many others that are addressing similar or same topics. Is it only the Pakistani population? Is it Karachi?

17. General comments: Please correct grammar and spelling.

18. Please submit a similarity index.

Reviewer #2: Title and Abstract:

• Title indicated study design

• Gives a brief about what the article is about, significance of the study while the aim is stated clearly.

Introduction :

• Line 77 and 78 remove Author initials

• Literature review was not clear on the associated factorS impacting the smile perception.

• Rationale clearly stated

• Aim clearly mentioned

• Objectives not stated, please mention them

Methods:

• Study population not clearly described

• Study setting not explained

• Inclusion and exclusion criteria not clearly stated

• No clear description of how the individual variables were measured, where the study questionnaire was adapted from and was it validated??

Results:

• What was the response rate and explain why

• There is inconsistency on the mentioned sample size of 386 and the result mention of a total of 864 participants

• On the tables, use small letter n, for the study proportions and a small letter p for p value

• General editing required

• No clear mention of the reference group in the regression analysis

Discussion:

• Some statements need references, e.g. line 216 and 218

• Sample size mentioned in the limitations, explain the limitation and mention how to overcome it.

• Statement on line 259, please explain. Did you control for confounding in the regression analysis??

General comments:

• Good study with important findings

• The article needs some editing.

**********

what does this mean?). If published, this will include your full peer review and any attached files.

If you choose “no”, your identity will remain anonymous but your review may still be made public.

**Do you want your identity to be public for this peer review?**  For information about this choice, including consent withdrawal, please see our Privacy Policy

Reviewer #1: **Yes:** Prof Vesna Karic

Reviewer #2: **Yes:** Dr CB MALAU

**********

[NOTE: If reviewer comments were submitted as an attachment file, they will be attached to this email and accessible via the submission site. Please log into your account, locate the manuscript record, and check for the action link "View Attachments". If this link does not appear, there are no attachment files.]

To ensure your figures meet our technical requirements, please review our figure guidelines: https://journals.plos.org/plosone/s/figures

You may also use PLOS’s free figure tool, NAAS, to help you prepare publication quality figures: https://journals.plos.org/plosone/s/figures#loc-tools-for-figure-preparation.

NAAS will assess whether your figures meet our technical requirements by comparing each figure against our figure specifications.

Attachment Submitted filename: Reviewed PONE-D-25-45043 (1).pdf

Attachment Submitted filename: Article review Plos one 21 NOV 2025.pdf

# Author comment: Author response to Decision Letter 1

**DOI:** 10.1371/journal.pone.0357126.r003

29 Jan 2026

ONE-D-25-45043

Dental aesthetic self-perception, smile satisfaction and associated factors among adults: a cross-sectional study

PLOS ONE

Journal Requirements:

When submitting your revision, we need you to address these additional requirements.

1. Please ensure that your manuscript meets PLOS ONE's style requirements, including those for file naming. The PLOS ONE style templates can be found at https://journals.plos.org/plosone/s/file?id=wjVg/PLOSOne_formatting_sample_main_body.pdf and https://journals.plos.org/plosone/s/file?id=ba62/PLOSOne_formatting_sample_title_authors_affiliations.pdf

Response: We have checked, and our manuscript follows PLOS ONE's style requirements.

2. PLOS requires an ORCID iD for the corresponding author in Editorial Manager on papers submitted after December 6th, 2016. Please ensure that you have an ORCID iD and that it is validated in Editorial Manager. To do this, go to ‘Update my Information’ (in the upper left-hand corner of the main menu), and click on the Fetch/Validate link next to the ORCID field. This will take you to the ORCID site and allow you to create a new iD or authenticate a pre-existing iD in Editorial Manager.

Response: The corresponding author’s ORCID iD has been validated.

3. Please include a complete copy of PLOS’ questionnaire on inclusivity in global research in your revised manuscript. Our policy for research in this area aims to improve transparency in the reporting of research performed outside of researchers’ own country or community. The policy applies to researchers who have travelled to a different country to conduct research, research with Indigenous populations or their lands, and research on cultural artefacts. The questionnaire can also be requested at the journal’s discretion for any other submissions, even if these conditions are not met. Please find more information on the policy and a link to download a blank copy of the questionnaire here: https://journals.plos.org/plosone/s/best-practices-in-research-reporting. Please upload a completed version of your questionnaire as Supporting Information when you resubmit your manuscript.

Response: The questionnaire has been attached as supporting information.

4. We note that there is identifying data in the Supporting Information file <S1 File.csv>.

Response: All identifying information has been anonymized.

5. If the reviewer comments include a recommendation to cite specific previously published works, please review and evaluate these publications to determine whether they are relevant and should be cited. There is no requirement to cite these works unless the editor has indicated otherwise.

Response: Thanks for pointing this out.

Additional Editor Comments:

Major methodological corrections. See details in the Reviewers' reports.

Response: All reviewers’ suggestions and comments have been incorporated into the manuscript.

Reviewers' comments:

Reviewer's Responses to Questions

Comments to the Author

1. Is the manuscript technically sound, and do the data support the conclusions?

The manuscript must describe a technically sound piece of scientific research with data that supports the conclusions. Experiments must have been conducted rigorously, with appropriate controls, replication, and sample sizes. The conclusions must be drawn appropriately based on the data presented.

Reviewer #1: Yes

Reviewer #2: Yes

Response: Thank you for your comments.

2. Has the statistical analysis been performed appropriately and rigorously?

Reviewer #1: Yes

Reviewer #2: Yes

Response: Thank you for your comments.

3. Have the authors made all data underlying the findings in their manuscript fully available?

The PLOS Data policy requires authors to make all data underlying the findings described in their manuscript fully available without restriction, with rare exception (please refer to the Data Availability Statement in the manuscript PDF file). The data should be provided as part of the manuscript or its supporting information, or deposited to a public repository. For example, in addition to summary statistics, the data points behind means, medians and variance measures should be available. If there are restrictions on publicly sharing data—e.g. participant privacy or use of data from a third party—those must be specified.

Reviewer #1: Yes

Reviewer #2: Yes

Response: Thank you for your comments.

4. Is the manuscript presented in an intelligible fashion and written in standard English?

PLOS ONE does not copyedit accepted manuscripts, so the language in submitted articles must be clear, correct, and unambiguous. Any typographical or grammatical errors should be corrected at revision, so please note any specific errors here.

Reviewer #1: No

Reviewer #2: Yes

Response: Thank you for your comments. The whole manuscript has been checked for spelling and grammar. General editing has been done throughout the manuscript. Additionally, we have used Grammarly to check the manuscript and improve sentence structures.

5. Review Comments to the Author

Please use the space provided to explain your answers to the questions above. You may also include additional comments for the author, including concerns about dual publication, research ethics, or publication ethics. (Please upload your review as an attachment if it exceeds 20,000 characters)

Reviewer #1: Dear authors

This very important article has a very important message.

Response: Thank you very much for liking the research idea.

This article could be published after following major corrections:

1. Title: Considering adding a pilot study since the sample can be much larger for Pakistan.

Response: Thank you for this valuable comment. We respectfully submit that the term “pilot study” may not be appropriate for this manuscript. Pilot studies are typically conducted to test feasibility, instruments, or procedures and are not primarily intended to generate substantive findings. In contrast, the present study was designed as a full cross-sectional investigation with a priori sample size calculation, a large final sample (n = 854), and inferential statistical analyses, including multivariable logistic regression.

Although Pakistan has a large population, the adequacy of a study sample is determined by study design, objectives, and statistical power rather than national population size alone. The final sample exceeded the minimum required sample size and provided sufficient power to address the study objectives.

We acknowledge the reviewer’s concern regarding generalizability. To address this, we have clearly stated in the limitations section that the study was conducted in a single metropolitan city using convenience sampling, which may limit national representativeness. We believe this clarification more accurately reflects the scope of the study than labeling it as a pilot.

2. Abstract: Methods: The online questionnaire link was distributed via email and social media apps. – Please specify to whom the questioners have been distributed. For example, the population of Karachi.

Response: Thank you for pointing this out. We have added this point to the manuscript. The questionnaire was distributed among the population of Karachi.

3. Abstract: Results: Eight hundred fifty-four individuals participated in the study, comprising 276 males (32.3%) and 578 females (67.7%). – Please specify to which population. Is it worldwide or just Pakistan? Is it Karachi?

Response: Thank you for pointing this out. We have added this point to the manuscript. This was the population of Karachi.

4. Introduction: A study conducted in Saudi Arabia in 2023 revealed that the most common dental issue affecting people's satisfaction was inadequate filling of front teeth [9]. – Please replace the word filling with restoration.

Response: Thank you for pointing this out. We have replaced the word filling with restoration.

5. Introduction: Multiple studies, including one by Nazir R in 2014 and another by Gassem AA in 2022, indicated that malocclusion, particularly in the front teeth, negatively impacts an individual's personality and self-esteem [10,11]. – Don’t use authors' names. Please remove authors' names and rewrite the sentence. Please correct the entire article.

Response: Thank you for pointing this out. We have removed the authors’ names.

6. Introduction: When malocclusion, especially in the front teeth, is present, it triggers negative social responses, ultimately leading to a reduced self-image [10,11]. – Please rewrite the sentence. Please change the front teeth to anterior teeth. For example, upper and lower anterior teeth.

Response: Thank you for pointing this out. We have replaced the word front with anterior and the sentence has been rephrased.

7. Discussion: A significant majority of the participants expressed admiration for another person's smile. This finding highlights the widespread recognition and appreciation of the positive effect of a warm and genuine smile on interpersonal interactions. – Please specify that this is a current study result.

Response: Thank you for pointing this out. We have modified the sentence to include the current study’s results.

8. Discussion: The results parallel those of a Saudi study – Please rewrite the sentence.

Response: Thank you for pointing this out. We have rewritten the sentence.

9. Discussion: Thirty-nine percent of participants reported frequently or occasionally avoiding smiling when their photographs were taken. Notably, there was no difference in opinion between the sexes. Participants provided various reasons for this behavior, including concerns regarding the appearance of their teeth in terms of size, shape, and color, as well as misalignments and gaps. – Please specify that this is a current study result. Please correct the word color.

Response: Thank you for pointing this out. We have modified the sentence to include the current study’s results. We have corrected the word color.

10. Discussion: Our research shows that most individuals surveyed agree on the significant influence a pleasant smile can have on a person's overall demeanor. – Don’t use personal language(our). Rather start the sentence with the current study. Please correct spelling for a word demeanor.

Response: Thank you for pointing this out. We have modified the sentence. We have corrected the spelling of the word demeanour.

11. Discussion: Please state the importance of the study. Please explain what value this study adds to already available information.

Response: Thank you for pointing this out. The following paragraph has been added to the manuscript.

Importance of the study

The study is important because it provides population-based data regarding the perception of adults in terms of their dental aesthetics and their satisfaction with their smiles and how these perceptions relate to their psychosocial well-being in an urban setting in South Asia. Most of the published studies on the topic are from Western or Middle Eastern backgrounds. The general adult population in Pakistan, and particularly in Karachi, has only limited data. Therefore, the current study fills this gap and provides culturally and regionally applicable information on the issue of smile perception and satisfaction in adults.

The research contributes to the current body of knowledge in a number of significant aspects. It emphasizes that over one-third of adults are unhappy with their smiles, but sociodemographic factors of age, sex, education, and occupation did not have a strong connection with smile satisfaction. It is important to note that the present study results defy the belief in the demographic factors of smile satisfaction, and highlight the predominance of the personal perceptions and subjective experience that is usually under-researched in dental public health studies. The present research supports and builds on previous research regarding the psychosocial significance of smiling, as it indicates that dental aesthetics may not be regarded as a cosmetic factor only, but rather as a part of a general QoL. The present study findings can be used to improve the relationship between clinical evaluation and patient expectations and assist in better patient-focused treatment planning and give priority to affordable aesthetic and orthodontic services in resource-constrained settings.

12. Implication of the study: Our results indicate several exciting findings. - Don’t use personal language(our).

Response: Thank you for pointing this out. We have modified the sentence.

13. Implication of the study: Our findings have significant implications for all stakeholders. - Don’t use personal language(our).

Response: Thank you for pointing this out. We have modified the sentence.

14. Conclusion: Please rewrite the conclusion.

Response: A few points in the conclusion section have been rewritten.

15. General comments: Discussion: Please add references.

Response: Thank you for pointing this out. Five new references have been added (four from 2025 and one from 2026 published studies).

16. General comments: Discussion: It is very important to highlight why this study is important and different as many others that are addressing similar or same topics. Is it only the Pakistani population? Is it Karachi?

Response: Thank you for pointing this out. A section on the importance of the present study and its value to the existing literature has been added to the discussion section.

17. General comments: Please correct grammar and spelling.

Response: Thank you for pointing this out. The whole manuscript has been checked for spelling and grammar. Additionally, we have used Grammarly to check the manuscript.

18. Please submit a similarity index.

Response: The similarity report is attached with the revised manuscript.

Reviewer #2: Title and Abstract:

• Title indicated study design

• Gives a brief about what the article is about, significance of the study while the aim is stated clearly.

Response: Thank you

Introduction :

• Line 77 and 78 remove Author initials

Response: Thank you for the suggestions. The authors’ names have been deleted.

• Literature review was not clear on the associated factors impacting the smile perception.

Response: Thank you for the comments. Please check line No 68-83. Several research articles have been mentioned regarding associated factors impacting the smile perception. Few more studies have been mentioned in the discussion section.

• Rationale clearly stated

Response: Thank you

• Aim clearly mentioned

Response: Thank you

• Objectives not stated, please mention them

Response: Thank you for pointing this out. We have rephrased the sentence “Consequently, this study was designed with the objectives to explore dental aesthetic self-perception, smile satisfaction, and related factors among a sample of the adult general population in Karachi, Pakistan.”

Methods:

• Study population not clearly described

Response: Thank you for the comments. The study population has been described in the revised manuscript. Please check lines No. 118-125.

• Study setting not explained

Response: Thank you for the comments. The study setting has been explained in the revised manuscript. Please check lines No. 102-109.

• Inclusion and exclusion criteria not clearly stated

Response: Thank you for the comments. The inclusion and exclusion criteria have been clearly stated in the revised manuscript. Please check lines No. 118-125.

• No clear description of how the individual variables were measured, where the study questionnaire was adapted from and was it validated??

Response: Thank you for the comments. The description of individual variables measurement have mentioned in the revised manuscript. Please check lines No. 127-30.

Two references for study questionnaire have already been given. Yes, it was validated.

“The researchers designed an online questionnaire and included a few questions from already published, well-known, validated questionnaires, namely, the "Aesthetic Component (AC) of the Index of Orthodontic Treatment Need (IOTN)" and the "Psychosocial Impact of Dental Aesthetics Questionnaire (PIDAQ)" [17,18]. Two subject experts checked the content and construct validity of the questionnaire, and modifications were made accor

Attachment Submitted filename: Response to reviewers comments.docx

# Peer review: Decision Letter 1

**DOI:** 10.1371/journal.pone.0357126.r004

**Authors:** Yolanda Malele-Kolisa

20 Mar 2026

Dear Dr. Baig,

Thank you for submitting your manuscript to PLOS ONE. After careful consideration, we feel that it has merit but does not fully meet PLOS ONE’s publication criteria as it currently stands. Therefore, we invite you to submit a revised version of the manuscript that addresses the points raised during the review process.

Please submit your revised manuscript by May 04 2026 11:59PM. If you will need more time than this to complete your revisions, please reply to this message or contact the journal office at plosone@plos.org. When you're ready to submit your revision, log on to https://www.editorialmanager.com/pone/ and select the 'Submissions Needing Revision' folder to locate your manuscript file.

- A letter that responds to each point raised by the academic editor and reviewer(s). You should upload this letter as a separate file labeled 'Response to Reviewers'.
- A marked-up copy of your manuscript that highlights changes made to the original version. You should upload this as a separate file labeled 'Revised Manuscript with Track Changes'.
- An unmarked version of your revised paper without tracked changes. You should upload this as a separate file labeled 'Manuscript'.

If applicable, we recommend that you deposit your laboratory protocols in protocols.io to enhance the reproducibility of your results. Protocols.io assigns your protocol its own identifier (DOI) so that it can be cited independently in the future. For instructions see: https://journals.plos.org/plosone/s/submission-guidelines#loc-laboratory-protocols. Additionally, PLOS ONE offers an option for publishing peer-reviewed Lab Protocol articles, which describe protocols hosted on protocols.io. Read more information on sharing protocols at https://plos.org/protocols?utm_medium=editorial-email&utm_source=authorletters&utm_campaign=protocols.

We look forward to receiving your revised manuscript.

Kind regards,

Yolanda Malele-Kolisa, BDS, MPH, MDent, PhD

Academic Editor

PLOS One

**Journal Requirements:**

1. If the reviewer comments include a recommendation to cite specific previously published works, please review and evaluate these publications to determine whether they are relevant and should be cited. There is no requirement to cite these works unless the editor has indicated otherwise.

2. Please review your reference list to ensure that it is complete and correct. If you have cited papers that have been retracted, please include the rationale for doing so in the manuscript text, or remove these references and replace them with relevant current references. Any changes to the reference list should be mentioned in the rebuttal letter that accompanies your revised manuscript. If you need to cite a retracted article, indicate the article’s retracted status in the References list and also include a citation and full reference for the retraction notice.

[Note: HTML markup is below. Please do not edit.]

Reviewers' comments:

Reviewer's Responses to Questions

**Comments to the Author**

Reviewer #1: All comments have been addressed

Reviewer #3: (No Response)

**********

2. Is the manuscript technically sound, and do the data support the conclusions?

Reviewer #1: Yes

Reviewer #3: Partly

**********

3. Has the statistical analysis been performed appropriately and rigorously?

Reviewer #1: Yes

Reviewer #3: I Don't Know

**********

4. Have the authors made all data underlying the findings in their manuscript fully available?

The PLOS Data policy

Reviewer #1: Yes

Reviewer #3: No

**********

5. Is the manuscript presented in an intelligible fashion and written in standard English?

Reviewer #1: Yes

Reviewer #3: Yes

**********

**Reviewer #1:** (No Response)

**Reviewer #3:** The manuscript lacks essential and basic information that is standard in a scientific piece. The interpretation of the results and discussions thereof is not convincing, and the conclusion does not tie to the evidence presented. The authors should look at explaining why, how and when: answering these questions may help fill in the gaps.

Lastly formatting will be necessary, and all tables & Figures must be on the same page as the description.

All comments are made in the text in the manuscript.

**********

what does this mean?). If published, this will include your full peer review and any attached files.

If you choose “no”, your identity will remain anonymous but your review may still be made public.

**Do you want your identity to be public for this peer review?**  For information about this choice, including consent withdrawal, please see our Privacy Policy

Reviewer #1: **Yes:** Prof Vesna Karic

Reviewer #3: **Yes:** Meriting Thokoane

**********

[NOTE: If reviewer comments were submitted as an attachment file, they will be attached to this email and accessible via the submission site. Please log into your account, locate the manuscript record, and check for the action link "View Attachments". If this link does not appear, there are no attachment files.]

To ensure your figures meet our technical requirements, please review our figure guidelines: https://journals.plos.org/plosone/s/figures

You may also use PLOS’s free figure tool, NAAS, to help you prepare publication quality figures: https://journals.plos.org/plosone/s/figures#loc-tools-for-figure-preparation.

NAAS will assess whether your figures meet our technical requirements by comparing each figure against our figure specifications.

Attachment Submitted filename: Manuscript PLOS ONE MGT comments.docx

# Author comment: Author response to Decision Letter 2

**DOI:** 10.1371/journal.pone.0357126.r005

9 Apr 2026

PONE-D-25-45043R1

Dental aesthetic self-perception, smile satisfaction and associated factors among adults: a cross-sectional study

PLOS One

Reviewers' comments:

Reviewer's Responses to Questions

Comments to the Author

1. If the authors have adequately addressed your comments raised in a previous round of review and you feel that this manuscript is now acceptable for publication, you may indicate that here to bypass the “Comments to the Author” section, enter your conflict of interest statement in the “Confidential to Editor” section, and submit your "Accept" recommendation.

Reviewer #1: All comments have been addressed

Response: Thank you

Reviewer #3: (No Response)

Response:

2. Is the manuscript technically sound, and do the data support the conclusions?

The manuscript must describe a technically sound piece of scientific research with data that supports the conclusions. Experiments must have been conducted rigorously, with appropriate controls, replication, and sample sizes. The conclusions must be drawn appropriately based on the data presented.

Reviewer #1: Yes

Response: Thank you

Reviewer #3: Partly

Response: We have modified the manuscript as suggested. Hopefully, now the manuscript is technically sound.

3. Has the statistical analysis been performed appropriately and rigorously?

Reviewer #1: Yes

Response: Thank you

Reviewer #3: I Don't Know

4. Have the authors made all data underlying the findings in their manuscript fully available?

Reviewer #1: Yes

Response: Thank you

Reviewer #3: No

Response: We have uploaded the CSV file of the data. So all the data given in the manuscript is freely available.

5. Is the manuscript presented in an intelligible fashion and written in standard English?

PLOS ONE does not copyedit accepted manuscripts, so the language in submitted articles must be clear, correct, and unambiguous. Any typographical or grammatical errors should be corrected at revision, so please note any specific errors here.

Reviewer #1: Yes

Response: Thank you

Reviewer #3: Yes

Response: Thank you

6. Review Comments to the Author

Please use the space provided to explain your answers to the questions above. You may also include additional comments for the author, including concerns about dual publication, research ethics, or publication ethics. (Please upload your review as an attachment if it exceeds 20,000 characters)

Reviewer #1: (No Response)

Reviewer #3: The manuscript lacks essential and basic information that is standard in a scientific piece. The interpretation of the results and discussions thereof is not convincing, and the conclusion does not tie to the evidence presented. The authors should look at explaining why, how and when: answering these questions may help fill in the gaps.

Lastly formatting will be necessary, and all tables & Figures must be on the same page as the description.

All comments are made in the text in the manuscript.

Response: Thank you for comments. We appreciate reviewer’s keen observation and constructive comments. We have modified the manuscript and addressed all points suggested by the reviewer. Sometimes, it is difficult to keep figure or table on the same page where description is given because of the continuity of the text or length of the table.

Please add references and percentages of how much each one was reported to be prominent.

Response: The following reference and percentages have been added.

A study reported that the eyes contribute 25%, the mouth 18%, face shape 15%, Skin 12%, and the nose 6% to the overall assessment of facial attractiveness (Ibrahim et al., 2024).

If the use of adolescent here is meant to indicate low...rather refer toit as low self esteem.

Response: Thank you for the suggestion. We have corrected it.

Is this limited to adults only? Do children and young adults not suffer the same fate?

Response: You are right. Thank you for pointing this out. We have rephrased it. Actually, we have used social interactions, quality of life, which is why we used adults.

Except in this study participants will be assessing their own smiles make this statement relevant to the discussion.

Response: Thank you for the suggestion. We have shifted this statement from the introduction to discussion section.

What is filling referring to here: restorations OR space-diastemata?

Response: Here, filling is referring to gap or space between two or more teeth,

Please provide a reference for this statement.

Response: The following references have been written. The reference numbers 10 and 11.

“The way individuals perceive their appearance significantly influences their personality and can trigger negative social responses that may ultimately lead to a reduced self-image [10,11].”

This will also help manage patients, expectations.

Response: Thank for the suggestions. We have incorporated this phrase into the sentence.

Please name the related factors.

Response: The names of the related factors have been included.

This study aimed to explore dental aesthetic self-perception, smile satisfaction, and related sociodemographic factors, including age, gender, marital status, education, and occupation, among a sample of the general adult population in Karachi, Pakistan.

State clearly who were the participants and how they were chosen &/or contacted to participate on the online survey.

Response: The study participants were recruited from the general population of Karachi via an online questionnaire. The questionnaire link was distributed via email and social media platforms, including WhatsApp, Facebook, Instagram, and Twitter, and two reminders were also sent at one-week intervals. Additionally, the researchers used their classmates, friends, and family contacts for recruiting study participants.

Were the participants not completing the survey? How long were they given?

Response: The researchers used their classmates, friends, and family contacts to recruit more study participants. The questionnaire link was distributed via email and social media platforms, including WhatsApp, Facebook, Instagram, and Twitter, and two reminders were also sent at one-week intervals.

Is this urban/ rural/ peri-urban?

Response: Karachi is a cosmopolitan city and the most developed urban area in Pakistan. This sentence has been included in the method section.

How was this sample size determined?

Response: The sample size determination method has been included in the sentence.

The sample size, 386, was determined before the start of the study using the Raosoft sample size calculator (http://www.raosoft.com/samplesize.html. Accessed on October 20, 2022) with a 5% margin of error and a 95% confidence level, using a population of 20 million for Karachi. The sample size was increased to enhance the survey's generalizability.

When was this accessed: web address & date?

Response: The web address & date have been included in the sentence.

The sample size, 386, was determined before the start of the study using the Raosoft sample size calculator (http://www.raosoft.com/samplesize.html. Accessed on October 20, 2022) with a 5% margin of error and a 95% confidence level, using a population of 20 million for Karachi. The sample size was increased to enhance the survey's generalizability. Generally, the larger sample improves the survey's precision and reliability and enables better subgroup analyses.

How were these identified...what made them relevant for your study?

Response: Two subject-matter experts assessed the content and construct validity of the questionnaire, and modifications were made based on their suggestions.

Please cite the software correctly and in full.

Response: Data were analyzed using IBM Statistical Package for the Social Sciences (SPSS) for Windows, Version 26.0 (IBM Corp., Armonk, NY, USA).

When did it become this and not 386?

Response: Thank you for this important comment. The calculated sample size of 384 represented the minimum required sample for adequate statistical precision. We collected 854 responses because larger sample sizes are acceptable and can strengthen survey-based studies. The increased sample improved the precision and reliability of the estimates, reduced sampling error, and allowed for better subgroup analyses. In addition, questionnaire-based studies often anticipate incomplete or invalid responses; therefore, collecting more responses helps ensure sufficient analyzable data. Since eligible responses continued to be received during the study period, all complete responses were included to enhance the representativeness and robustness of the findings.

Could not find figure 1.

Response: If figures are missing, we apologize. Actually, both figures were uploaded separately as required by the journal. Usually, these are present at the end of the PDF file that’s sent to the reviewer.

Could not locate figure 2

Response: If figures are missing, we apologize. Actually, both figures were uploaded separately as required by the journal. Usually, these are present at the end of the PDF file that’s sent to the reviewer.

Please show the different findings from the various studies in percentages within text

Response: Two dissimilar references have been added.

Please add percentages from each study in-text.

Response: The percentages have been added.

Please add relevant percentages.

Response: The relevant percentages have been added.

References please.

Response: The references have been added.

Please quantify the ‘significant majority’.

Response: The percentage has been added to quantify the ‘significant majority’.

Is it genuine smile OR being able to smile?

Response: The sentence has been modified by removing word ‘genuine’.

Observed between genders, or age-groups, or level of education?

Response: Actually, this is the overall agreement not between genders, or age-groups, or level of education

Overall level of agreement (85.9%) observed in this study regarding the impact of tooth color on smile attractiveness emphasizes the significance of tooth appearance.

State the percentage please

Response: The percentage has been added.

318(37,2%) versus 62,8%

Response: The percentage has been added

To ensure your figures meet our technical requirements, please review our figure guidelines: https://journals.plos.org/plosone/s/figures

Response: Yes figure meet the journal’s technical requirements

You may also use PLOS’s free figure tool, NAAS, to help you prepare publication quality figures: https://journals.plos.org/plosone/s/figures#loc-tools-for-figure-preparation.

NAAS will assess whether your figures meet our technical requirements by comparing each figure against our figure specifications.

Response: Yes, We have used NAAS for preparing ou figure meet the journal’s technical requirements

Attachment Submitted filename: Response_to_reviewers_comments_auresp_2.docx

# Peer review: Decision Letter 2

**DOI:** 10.1371/journal.pone.0357126.r006

**Authors:** Xin Sun

19 Jul 2026

Dear Dr. Baig,

Thank you for submitting your manuscript to PLOS ONE. After careful consideration, we feel that it has merit but does not fully meet PLOS ONE’s publication criteria as it currently stands. Therefore, we invite you to submit a revised version of the manuscript that addresses the points raised during the review process.

Please submit your revised manuscript by Aug 31 2026 11:59PM. If you will need more time than this to complete your revisions, please reply to this message or contact the journal office at plosone@plos.org. When you're ready to submit your revision, log on to https://www.editorialmanager.com/pone/ and select the 'Submissions Needing Revision' folder to locate your manuscript file.

- A letter that responds to each point raised by the academic editor and reviewer(s). You should upload this letter as a separate file labeled 'Response to Reviewers'.
- A marked-up copy of your manuscript that highlights changes made to the original version. You should upload this as a separate file labeled 'Revised Manuscript with Track Changes'.
- An unmarked version of your revised paper without tracked changes. You should upload this as a separate file labeled 'Manuscript'.

If you would like to make changes to your financial disclosure, please include your updated statement in your cover letter. Guidelines for resubmitting your figure files are available below the reviewer comments at the end of this letter.

If applicable, we recommend that you deposit your laboratory protocols in protocols.io to enhance the reproducibility of your results. Protocols.io assigns your protocol its own identifier (DOI) so that it can be cited independently in the future. For instructions see: https://journals.plos.org/plosone/s/submission-guidelines#loc-laboratory-protocols. Additionally, PLOS ONE offers an option for publishing peer-reviewed Lab Protocol articles, which describe protocols hosted on protocols.io. Read more information on sharing protocols at https://plos.org/protocols?utm_medium=editorial-email&utm_source=authorletters&utm_campaign=protocols.

As the corresponding author, your ORCID iD is verified in the submission system and will appear in the published article. PLOS supports the use of ORCID, and we encourage all coauthors to register for an ORCID iD and use it as well. Please encourage your coauthors to verify their ORCID iD within the submission system before final acceptance, as unverified ORCID iDs will not appear in the published article. *Only*  the individual author can complete the verification step; PLOS staff *cannot*  verify ORCID iDs on behalf of authors.

We look forward to receiving your revised manuscript.

Kind regards,

Xin Sun, PhD

Staff Editor

PLOS One

Journal Requirements:

If the reviewer comments include a recommendation to cite specific previously published works, please review and evaluate these publications to determine whether they are relevant and should be cited. There is no requirement to cite these works unless the editor has indicated otherwise.

Please review your reference list to ensure that it is complete and correct. If you have cited papers that have been retracted, please include the rationale for doing so in the manuscript text, or remove these references and replace them with relevant current references. Any changes to the reference list should be mentioned in the rebuttal letter that accompanies your revised manuscript. If you need to cite a retracted article, indicate the article’s retracted status in the References list and also include a citation and full reference for the retraction notice.

[Note: HTML markup is below. Please do not edit.]

Reviewers' comments:

Reviewer's Responses to Questions

**Comments to the Author**

Reviewer #1: All comments have been addressed

Reviewer #3: All comments have been addressed

Reviewer #4: (No Response)

**********

2. Is the manuscript technically sound, and do the data support the conclusions?

Reviewer #1: Yes

Reviewer #3: Yes

Reviewer #4: Partly

**********

3. Has the statistical analysis been performed appropriately and rigorously?

Reviewer #1: Yes

Reviewer #3: I Don't Know

Reviewer #4: N/A

**********

4. Have the authors made all data underlying the findings in their manuscript fully available?

The PLOS Data policy

Reviewer #1: Yes

Reviewer #3: Yes

Reviewer #4: No

**********

5. Is the manuscript presented in an intelligible fashion and written in standard English?

Reviewer #1: Yes

Reviewer #3: Yes

Reviewer #4: Yes

**********

Reviewer #1: Congratulations on your article. Please correct following before publication:

1. Malocclusion, particularly in the front teeth, negatively impacts an individual's personality and self-esteem [10,11], - Add full stop.

Reviewer #3: (No Response)

Reviewer #4: PEER REVIEW REPORT

Manuscript: Dental Aesthetic Self-Perception, Smile Satisfaction , and Associated Factors Among the General Adult Population in Karachi, Pakistan

Study Design: Cross-sectional Survey

Setting: LCMD, Karachi

Period: November 2022-March 2023

Reviewer : Kinder Modisha

1. SUMMARY OF THE MANUSCRIPT

This cross-sectional study investigated dental aesthetic self-perception, smile satisfaction, and associated psychosocial factors in 854 adults recruited from Karachi, Pakistan. Using a self-administered online questionnaire, distributed via email and social media between November 2022 and March 2023. The authors report that 37.2% of participants were dissatisfied with their smiles, that the majority of the participants linked smiling to mental and social well-being, and that there was no significant associations found between sociodemographic variables and smile satisfaction in binary logistic regression.

2. STRENGTHS

• The sample size was adequate (n=854)

• The study addresses clinically and psychologically topics that are relevant, which then will mean that dental care will be patient-centred in their country’s context.

• The research question is clearly articulated and the study fills a real gap in the published literature on smile satisfaction in Pakistan population

• Inclusion of gender-disaggregated analysis provides useful insight into different perceptions of smile aesthetics

3. MAJOR CONCERNS

3.1 Sampling Strategy and Selection Bias

With the study relying on convenience sampling via social media and email, it introduces an element of selection bias. Only those with better digital literacy, and stronger health awareness are overrepresented in online surveys. This will be problematic in a city like Karachi, which has a diverse socio-economic landscape, with the lager population group may lack consistent access to internet.

3.2 Absence of a Validated Instrument

The manuscript does not state whether the questionnaire was validated or adapted from an established, validated instrument. Dental aesthetics self-perception research commonly use validated tools such as the Oral Aesthetics Subjective Impact Scale (OASIS)

3.3 Statistical Reporting Errors

Several statistical inconsistencies are present in the abstract and results:

• The p-value for gender analysis of tooth colour is reported as “p<0.034” This is non-standard. If the p value is already below 0.05 then the correct reporting format should be “p=0.034”

• Rounding of percentages is not consistent: the author rounded of 69.67% to 69.7% (to those agreed that smile can impact mental , emotional and social wellbeing. But then rounded off 77,87% to 77,95% which the mumber are not the same

3.4 No mention of Ethical Approval and Informed Consent Documentation

There is no mention of ethical approval from the relevant board or Ethics Committee, nor is there any mention of informed consent procedures for online participation

4. MINOR CONCERNS

4.1 Introduction

The introduction as written in the abstract does not contextualise the study within the existing literature. No reference to any study in relation to smile satisfaction or dental aesthetics

4.2 Methods and Results

• Inclusion and Exclusion criteria are not described

• The gender breakdown is notable way to skew, 32.3% males, 67,7% females

4.3 Discussion And Conclusion

• The claim that more that 50% of subjects expressed contentment with their smiles is inconsistence with the finding that 37.2% were dissatisfied.

• The discussion should compare findings with similar studies from South of Asia, Middle East and other comparable lower-middle income country context

5. REQUIRED ACTIONS AD RECOMMENDATIONS

a) Provide IRB/Ethics Committee approval number and informed consent protocol details

b) Validate or cite the validation status of the Questionnaire used

c) Correct the p-value reporting error (p,0.034 …..p=0.034 and the percentage rounding off)

d) State the inclusion/exclusion criteria clearly in the Methods section

e) Provide a sample size justification or power calculation

f) Discuss the selection criteria bias from internet -based convenience sampling explicitly in the Limitations section

**********

what does this mean?). If published, this will include your full peer review and any attached files.

If you choose “no”, your identity will remain anonymous but your review may still be made public.

**Do you want your identity to be public for this peer review?**  For information about this choice, including consent withdrawal, please see our Privacy Policy

Reviewer #1: **Yes:** Prof Vesna Karic

Reviewer #3: No

Reviewer #4: **Yes:** Kinder Modisha

**********

[NOTE: If reviewer comments were submitted as an attachment file, they will be attached to this email and accessible via the submission site. Please log into your account, locate the manuscript record, and check for the action link "View Attachments". If this link does not appear, there are no attachment files.]

To ensure your figures meet our technical requirements, please review our figure guidelines: https://journals.plos.org/plosone/s/figures

You may also use PLOS’s free figure tool, NAAS, to help you prepare publication quality figures: https://journals.plos.org/plosone/s/figures#loc-tools-for-figure-preparation.

NAAS will assess whether your figures meet our technical requirements by comparing each figure against our figure specifications.

Attachment Submitted filename: Peer Review Report Dental Aesthetic.pdf

# Author comment: Author response to Decision Letter 3

**DOI:** 10.1371/journal.pone.0357126.r007

24 Jul 2026

Dear Editor and Reviewers,

We sincerely thank the editor and reviewers for their careful evaluation and constructive comments. We have revised the manuscript accordingly. All modifications are shown using the tracked changes format. In addition to the requested revisions, we corrected several internal inconsistencies and language problems to improve accuracy, transparency, and readability.

Sincerely,

Dr Javaria Baig

Journal Requirements:

If the reviewer comments include a recommendation to cite specific previously published works, please review and evaluate these publications to determine whether they are relevant and should be cited. There is no requirement to cite these works unless the editor has indicated otherwise.

Please review your reference list to ensure that it is complete and correct. If you have cited papers that have been retracted, please include the rationale for doing so in the manuscript text, or remove these references and replace them with relevant current references. Any changes to the reference list should be mentioned in the rebuttal letter that accompanies your revised manuscript. If you need to cite a retracted article, indicate the article’s retracted status in the References list and also include a citation and full reference for the retraction notice.

Response: The reference list was reviewed for consistency with the citations used in the manuscript. No reviewer-requested reference was added because no specific publication was recommended. No change to the reference list was required for the present revision. The authors should nevertheless complete the journal’s final database-based retraction check during submission.

A dedicated Data availability statement has been added to make explicit that all data underlying the findings are supplied as S1 File.

Reviewers' comments:

Comments to the Author

1. If the authors have adequately addressed your comments raised in a previous round of review and you feel that this manuscript is now acceptable for publication, you may indicate that here to bypass the “Comments to the Author” section, enter your conflict of interest statement in the “Confidential to Editor” section, and submit your "Accept" recommendation.

Reviewer #1: All comments have been addressed

Reviewer #3: All comments have been addressed

Reviewer #4: (No Response)

2. Is the manuscript technically sound, and do the data support the conclusions?

The manuscript must describe a technically sound piece of scientific research with data that supports the conclusions. Experiments must have been conducted rigorously, with appropriate controls, replication, and sample sizes. The conclusions must be drawn appropriately based on the data presented.

Reviewer #1: Yes

Reviewer #3: Yes

Reviewer #4: Partly

3. Has the statistical analysis been performed appropriately and rigorously?

Reviewer #1: Yes

Reviewer #3: I Don't Know

Reviewer #4: N/A

4. Have the authors made all data underlying the findings in their manuscript fully available?

The PLOS Data policy requires authors to make all data underlying the findings described in their manuscript fully available without restriction, with rare exception (please refer to the Data Availability Statement in the manuscript PDF file). The data should be provided as part of the manuscript or its supporting information, or deposited to a public repository. For example, in addition to summary statistics, the data points behind means, medians and variance measures should be available. If there are restrictions on publicly sharing data—e.g. participant privacy or use of data from a third party—those must be specified.

Reviewer #1: Yes

Reviewer #3: Yes

Reviewer #4: No

5. Is the manuscript presented in an intelligible fashion and written in standard English?

PLOS ONE does not copyedit accepted manuscripts, so the language in submitted articles must be clear, correct, and unambiguous. Any typographical or grammatical errors should be corrected at revision, so please note any specific errors here.

Reviewer #1: Yes

Reviewer #3: Yes

Reviewer #4: Yes

6. Review Comments to the Author

Please use the space provided to explain your answers to the questions above. You may also include additional comments for the author, including concerns about dual publication, research ethics, or publication ethics. (Please upload your review as an attachment if it exceeds 20,000 characters)

Reviewer 1

Congratulations on your article. Please correct following before publication:

1. Malocclusion, particularly in the front teeth, negatively impacts an individual's personality and self-esteem [10,11], - Add full stop.

Response: Thank you. We corrected the punctuation and also revised the surrounding sentences for grammar and clarity.

Reviewer 3

We thank Reviewer 3 for confirming that the previous comments were adequately addressed and for indicating that the manuscript is technically sound and clearly written. No additional revision was requested.

Reviewer 4

1. SUMMARY OF THE MANUSCRIPT

This cross-sectional study investigated dental aesthetic self-perception, smile satisfaction, and associated psychosocial factors in 854 adults recruited from Karachi, Pakistan. Using a self-administered online questionnaire, distributed via email and social media between November 2022 and March 2023. The authors report that 37.2% of participants were dissatisfied with their smiles, that the majority of the participants linked smiling to mental and social well-being, and that there was no significant associations found between sociodemographic variables and smile satisfaction in binary logistic regression.

Response: Thank you

2. STRENGTHS

• The sample size was adequate (n=854)

• The study addresses clinically and psychologically topics that are relevant, which then will mean that dental care will be patient-centred in their country’s context.

• The research question is clearly articulated and the study fills a real gap in the published literature on smile satisfaction in Pakistan population

• Inclusion of gender-disaggregated analysis provides useful insight into different perceptions of smile aesthetics

Response: Thank you

3. MAJOR CONCERNS

3.1 Sampling Strategy and Selection Bias

With the study relying on convenience sampling via social media and email, it introduces an element of selection bias. Only those with better digital literacy, and stronger health awareness are overrepresented in online surveys. This will be problematic in a city like Karachi, which has a diverse socio-economic landscape, with the lager population group may lack consistent access to internet.

Response: We agree. The method section now identifies the recruitment strategy explicitly as online convenience sampling. The limitations section has been substantially expanded to explain likely overrepresentation of younger, educated, digitally literate, health-aware, and internet-connected participants; underrepresentation of people with limited digital access; use of personal networks; single-city recruitment; and the fact that a large sample cannot remove selection bias.

3.2 Absence of a Validated Instrument

The manuscript does not state whether the questionnaire was validated or adapted from an established, validated instrument. Dental aesthetics self-perception research commonly use validated tools such as the Oral Aesthetics Subjective Impact Scale (OASIS)

Response: Thank you for identifying the need for clearer reporting. The study used a researcher-developed questionnaire incorporating selected questions and concepts from the IOTN Aesthetic Component and PIDAQ, rather than administering either complete instrument. The questionnaire was pilot-tested in 50 individuals and reviewed by two subject-matter experts for content relevance and clarity. The available validation evidence was limited to pilot testing and expert review; reliability coefficients and factor-analytic validation were not conducted. We now state this transparently in both the methods and limitations. OASIS was not used in this study; therefore, we have not presented the instrument as OASIS or added it as the source of the questionnaire.

3.3 Statistical Reporting Errors

Several statistical inconsistencies are present in the abstract and results:

• The p-value for gender analysis of tooth colour is reported as “p<0.034” This is non-standard. If the p value is already below 0.05 then the correct reporting format should be “p=0.034”

• Rounding of percentages is not consistent: the author rounded of 69.67% to 69.7% (to those agreed that smile can impact mental , emotional and social wellbeing. But then rounded off 77,87% to 77,95% which the mumber are not the same

Response: We agree. The exact p-value is now reported as p = 0.034, and 665/854 is consistently reported as 77.9%. We also standardized p-value spacing and percentage presentation. During this check, we found that the narrative statements claiming consistently higher endorsement among females did not accurately reflect the category percentages in Table 3. We therefore revised the text to report significant overall gender differences in response distributions without assigning an unsupported direction.

3.4 No mention of Ethical Approval and Informed Consent Documentation

There is no mention of ethical approval from the relevant board or Ethics Committee, nor is there any mention of informed consent procedures for online participation

Response: Ethical approval and consent information were present in the submitted Methods, but we agree that the procedure could be stated more explicitly. We have strengthened the description by retaining the LCMD Institutional Review Board reference number (IRB/D-000046/22) and explaining the pre-questionnaire information and consent statement, voluntary participation, electronic informed consent through proceeding and submission, anonymity, and use of the data for research publication.

4. MINOR CONCERNS

4.1 Introduction

The introduction as written in the abstract does not contextualise the study within the existing literature. No reference to any study in relation to smile satisfaction or dental aesthetics

Response: We agree. A concise contextual sentence has been added to the abstract, stating that dental aesthetic self-perception can influence self-confidence and psychosocial well-being and that evidence from Pakistan remains limited. The abstract objective has also been made more specific.

4.2 Methods and Results

• Inclusion and Exclusion criteria are not described

Response: The criteria were present in the original Methods but were embedded within the recruitment paragraph. We have reorganized and clarified them. Eligible participants were adults aged 18 years or older, male or female, and residents of Karachi. Exclusion criteria were previous orthodontic treatment and dental or facial injuries, conditions, or craniofacial anomalies such as cleft lip or palate.

• The gender breakdown is notable way to skew, 32.3% males, 67,7% females

Response: We agree. The unequal gender distribution is now explicitly acknowledged as a limitation affecting gender comparisons and generalizability. We also removed overinterpretation of gender differences from the results, discussion, and implications sections.

4.3 Discussion And Conclusion

• The claim that more that 50% of subjects expressed contentment with their smiles is inconsistence with the finding that 37.2% were dissatisfied.

Response: The two values are complementary: 536/854 participants (62.8%) were satisfied and 318/854 (37.2%) were dissatisfied. To avoid ambiguity, we replaced the phrase “more than 50%” with the exact value, 62.8%, in the abstract and conclusion.

• The discussion should compare findings with similar studies from South of Asia, Middle East and other comparable lower-middle income country context

Response: We agree. The comparative discussion has been consolidated and expanded using studies already cited in the manuscript. The revised text compares the present 62.8% satisfaction estimate with findings from Saudi Arabia, Vietnam, Malaysia, and Serbia, and additionally notes evidence from Pakistan and northern India regarding the psychosocial relevance of dental aesthetics and perceived orthodontic treatment need. Differences in age, culture, populations, sampling methods, and instruments are discussed as possible explanations for variation.

5. REQUIRED ACTIONS AD RECOMMENDATIONS

a) Provide IRB/Ethics Committee approval number and informed consent protocol details

Response: Provided

b) Validate or cite the validation status of the Questionnaire used

Response: Validation explained

c) Correct the p-value reporting error (p,0.034 …..p=0.034 and the percentage rounding off)

Response: all p-values have been corrected.

d) State the inclusion/exclusion criteria clearly in the Methods section

Response: Inclusion exclusion criteria has been explained.

e) Provide a sample size justification or power calculation

Response: sample size justification provided.

f) Discuss the selection criteria bias from internet -based convenience sampling explicitly in the Limitations section

Response: Selection bias has been mentioned in the limitations.

Additional accuracy and language corrections

• Corrected the female percentage for noticing the nose first from 3.6% to 6.2% because 36/578 = 6.2%; the frequency count was unchanged.

• Corrected typographical and grammatical errors throughout the abstract, Methods, Results, and Discussion.

• Replaced imprecise or unsupported interpretations with wording that remains within the cross-sectional, self-reported data.

• Standardized terminology, including 'buccal corridor,' p-value formatting, and the presentation of frequencies and percentages.

7. PLOS authors have the option to publish the peer review history of their article (what does this mean?). If published, this will include your full peer review and any attached files.

If you choose “no”, your identity will remain anonymous but your review may still be made public.

Do you want your identity to be public for this peer review? For information about this choice, including consent withdrawal, please see our Privacy Policy.

Reviewer #1: Yes: Prof Vesna Karic

Reviewer #3: No

Reviewer #4: Yes: Kinder Modisha

Attachment Submitted filename: Pointwise response to reviewers comments.docx

# Peer review: Decision Letter 3

**DOI:** 10.1371/journal.pone.0357126.r008

**Authors:** Johari Yap Abdullah

12 Aug 2026

Dental aesthetic self-perception, smile satisfaction and associated factors among adults: a cross-sectional study

PONE-D-25-45043R3

Dear Dr. Baig,

We’re pleased to inform you that your manuscript has been judged scientifically suitable for publication and will be formally accepted for publication once it meets all outstanding technical requirements.

Within one week, you’ll receive an e-mail detailing the required amendments. When these have been addressed, you’ll receive a formal acceptance letter and your manuscript will be scheduled for publication.

An invoice will be generated when your article is formally accepted. Please note, if your institution has a publishing partnership with PLOS and your article meets the relevant criteria, all or part of your publication costs will be covered. Please make sure your user information is up-to-date by logging into Editorial Manager at Editorial Manager® and clicking the ‘Update My Information' link at the top of the page. For questions related to billing, please contact billing support.

If your institution or institutions have a press office, please notify them about your upcoming paper to help maximize its impact. If they’ll be preparing press materials, please inform our press team as soon as possible -- no later than 48 hours after receiving the formal acceptance. Your manuscript will remain under strict press embargo until 2 pm Eastern Time on the date of publication. For more information, please contact onepress@plos.org.

Kind regards,

Johari Yap Abdullah, B.S. & I.T, GradDip ICT, M.Sc, Ph.D.

Academic Editor

PLOS One

Additional Editor Comments (optional):

Reviewers' comments:

Reviewer's Responses to Questions

**Comments to the Author**

Reviewer #1: All comments have been addressed

**********

2. Is the manuscript technically sound, and do the data support the conclusions?

Reviewer #1: Yes

**********

3. Has the statistical analysis been performed appropriately and rigorously?

Reviewer #1: Yes

**********

4. Have the authors made all data underlying the findings in their manuscript fully available?

The PLOS Data policy

Reviewer #1: Yes

**********

5. Is the manuscript presented in an intelligible fashion and written in standard English?

Reviewer #1: Yes

**********

Reviewer #1: (No Response)

**********

what does this mean?). If published, this will include your full peer review and any attached files.

If you choose “no”, your identity will remain anonymous but your review may still be made public.

**Do you want your identity to be public for this peer review?**  For information about this choice, including consent withdrawal, please see our Privacy Policy

Reviewer #1: **Yes:** Prof Vesna Karic

**********

# Editor report: Acceptance letter

**DOI:** 10.1371/journal.pone.0357126.r009

**Authors:** Johari Yap Abdullah

PONE-D-25-45043R3

PLOS One

Dear Dr. Baig,

I'm pleased to inform you that your manuscript has been deemed suitable for publication in PLOS One. Congratulations! Your manuscript is now being handed over to our production team.

At this stage, our production department will prepare your paper for publication. This includes ensuring the following:

* All references, tables, and figures are properly cited

* All relevant supporting information is included in the manuscript submission,

* There are no issues that prevent the paper from being properly typeset

You will receive further instructions from the production team, including instructions on how to review your proof when it is ready. Please keep in mind that we are working through a large volume of accepted articles, so please give us a few days to review your paper and let you know the next and final steps.

Lastly, if your institution or institutions have a press office, please let them know about your upcoming paper now to help maximize its impact. If they'll be preparing press materials, please inform our press team within the next 48 hours. Your manuscript will remain under strict press embargo until 2 pm Eastern Time on the date of publication. For more information, please contact onepress@plos.org.

You will receive an invoice from PLOS for your publication fee after your manuscript has reached the completed accept phase. If you receive an email requesting payment before acceptance or for any other service, this may be a phishing scheme. Learn how to identify phishing emails and protect your accounts at https://explore.plos.org/phishing.

If we can help with anything else, please email us at customercare@plos.org.

Thank you for submitting your work to PLOS One and supporting open access.

Kind regards,

PLOS One Editorial Office Staff

on behalf of

Dr. Johari Yap Abdullah

Academic Editor

PLOS One
